# Human proximal tubular epithelial cell interleukin-1 receptor signalling triggers G2/M arrest and cellular senescence during hypoxic kidney injury

**DOI:** 10.1038/s41419-025-07386-6

**Published:** 2025-01-31

**Authors:** Kurt T. K. Giuliani, Purba Nag, Benjamin C. Adams, Xiangju Wang, Seokchan Hong, Anca Grivei, Rebecca L. Johnston, Nicola Waddell, Kenneth K. C. Ho, Yilin Tian, Muhammad Ali Khan, Chang Seong Kim, Monica S. Y. Ng, Glenda Gobe, Jacobus P. J. Ungerer, Josephine M. Forbes, Helen G. Healy, Andrew J. Kassianos

**Affiliations:** 1 Conjoint Internal Medicine Laboratory, Chemical Pathology, Pathology Queensland, Brisbane, QLD Australia; 2 Kidney Health Service, Royal Brisbane and Women’s Hospital, Brisbane, QLD Australia; 3 Faculty of Medicine, University of Queensland, Brisbane, QLD Australia; 4 Division of Rheumatology, Department of Internal Medicine, Asan Medical Centre, Seoul, Republic of Korea; 5 QIMR Berghofer Medical Research Institute, Brisbane, QLD Australia; 6 Kidney Disease Research Collaborative, Princess Alexandra Hospital and University of Queensland, Translational Research Institute, Brisbane, QLD Australia; 7 Department of Pharmacy, Bangabandhu Sheikh Mujibur Rahman Science and Technology University, Dhaka, Bangladesh; 8 Department of Internal Medicine, Chonnam National University Hospital, Gwangju, Republic of Korea; 9 Department of Internal Medicine, Chonnam National University Medical School, Gwangju, Republic of Korea; 10 Mater Research Institute, University of Queensland, Brisbane, QLD Australia; 11 Institute of Health and Biomedical Innovation/School of Biomedical Sciences, Queensland University of Technology, Brisbane, QLD Australia

**Keywords:** Translational research, End-stage renal disease

## Abstract

Hypoxia and interleukin (IL)-1β are independent mediators of tubulointerstitial fibrosis, the histological hallmark of chronic kidney disease (CKD). Here, we examine how hypoxia and IL-1β act in synergy to augment maladaptive proximal tubular epithelial cell (PTEC) repair in human CKD. Ex vivo patient-derived PTECs were cultured under normoxic (21% O_2_) or hypoxic (1% O_2_) conditions in the absence or presence of IL-1β and examined for maladaptive repair signatures. Hypoxic PTECs incubated with IL-1β displayed a discrete transcriptomic profile distinct from PTECs cultured under hypoxia alone, IL-1β alone or under normoxia. Hypoxia+IL-1β-treated PTECs had 692 ‘unique’ differentially expressed genes (DEGs) compared to normoxic PTECs, with ‘cell cycle’ the most significantly enriched KEGG pathway based on ‘unique’ down-regulated DEGs (including *CCNA2*, *CCNB1* and *CCNB2*). Hypoxia+IL-1β-treated PTECs displayed signatures of cellular senescence, with reduced proliferation, G2/M cell cycle arrest, increased p21 expression, elevated senescence-associated β-galactosidase (SA-β-gal) activity and increased production of pro-inflammatory/fibrotic senescence-associated secretory phenotype (SASP) factors compared to normoxic conditions. Treatment of Hypoxia+IL-1β-treated PTECs with either a type I IL-1 receptor (IL-1RI) neutralizing antibody or a senolytic drug combination, quercetin+dasatinib, attenuated senescent cell burden. In vitro findings were validated in human CKD bio-specimens (kidney tissue, urine), with elevated PTEC IL-1RI expression and senescence (SA-β-gal activity) detected in fibrotic kidneys and numbers of senescent (SA-β-gal^+^) urinary PTECs correlating with urinary IL-1β levels and severity of interstitial fibrosis. Our data identify a mechanism whereby hypoxia in combination with IL-1β/IL-1RI signalling trigger PTEC senescence, providing novel therapeutic and diagnostic check-points for restoring tubular regeneration in human CKD.

## Introduction

The global burden of chronic kidney disease (CKD) has risen dramatically in recent years, driven by an ageing population and the increased prevalence of diabetes and hypertension [1]. The pathobiological hallmark of CKD is fibrosis within the tubulointerstitial compartment [2, 3]. Tubulointerstitial fibrosis is underpinned by sustained inflammation and irreversible tubular cell loss within the hypoxic CKD microenvironment [4]. Defining the hypoxic-inflammatory signalling mechanisms that culminate in tubulointerstitial fibrosis is critical for developing novel therapeutics for precision targeting of CKD progression.

Proximal tubular epithelial cells (PTECs) are a key tubular cell population in CKD [5, 6]. PTECs are susceptible to the hypoxic-inflammatory CKD microenvironment due to their reliance on mitochondrial fatty acid oxidation as a preferred energy source [7]. The induction of PTEC maladaptive repair mechanisms (e.g., cell death or cell cycle arrest/senescence) in response to these injurious stimuli is a central mediator of tubular loss and CKD progression [8–11]. In response to hypoxia alone, human PTECs undergo regulated cell death (i.e., ferroptosis) and selectively trigger pro-inflammatory interleukin (IL)-1β production in tubulointerstitial dendritic cells (DCs) [12]. IL-1β is elevated in human fibrotic kidneys [5] and contributes to maladaptive CKD signalling by binding to its cognate type I IL-1 receptor (IL-1RI) expressed on both immune and parenchymal cell populations (e.g., PTECs) [13]. Stimulation of healthy human PTECs with IL-1β alone induces pathways of oxidative stress and fibrogenesis (e.g., elevated transforming growth factor (TGF)-β1 production) [14, 15]. However, the complex response of human PTECs to hypoxia in combination with IL-1β/IL-1RI signalling, a more physiological representation of the CKD tubulointerstitium, remains poorly defined.

In this study, we show hypoxia and IL-1β act in synergy to trigger cellular senescence of patient-derived primary PTECs (i.e., reduced expression of cell cycling transcripts, decreased cell proliferation, cell cycle arrest in G2/M, increased p21 expression and elevated senescence-associated β-galactosidase (SA-β-gal) activity). We provide first in situ*-*ex vivo evidence of this concept in human CKD, with: [i] increased PTEC IL-1RI expression and senescence (SA-β-gal activity) in fibrotic kidneys; and [ii] positive correlations between numbers of senescent (SA-β-gal^+^) urinary PTECs and both urinary IL-1β levels and severity of interstitial fibrosis. Our data point to PTEC senescence driven by complex signalling mechanisms within the hypoxic-inflammatory tubulointerstitium as a novel clinical target for therapeutic and diagnostic translation in human CKD.

## Methods

### Isolation and culture of human primary proximal tubular epithelial cells (PTECs)

Kidney cortical tissue was obtained with informed patient consent from the macroscopically healthy portion of tumour nephrectomies (*n* = 18 patients, none with co-morbidities of hypertension and/or diabetes mellitus), following approval by the Royal Brisbane and Women’s Hospital Human Research Ethics Committee (2002/011 and 2006/072). Kidney tissue was immediately divided for: (1) isolation and culture of human primary PTECs; (2) freezing in Tissue-Tek OCT compound (Sakura, Torrance, CA, USA) for immunofluorescence (IF) analysis; and (3) fixation in formalin for morphological assessment of interstitial fibrosis/tubular atrophy by kidney histopathologists in a blinded manner. Kidney tissue specimens displaying ≥5% interstitial fibrosis were deemed fibrotic, based on the Banff 97 working classification of kidney pathology [16]. Tissue specimens were grouped into ‘non-fibrotic (control)’ (*n* = 13; 12 male, 1 female; mean age 55.3 ± 12.8 years; mean estimated glomerular filtration rate (eGFR) 83.3 ± 8.2 ml/min/1.73m^2^) or ‘fibrotic’ cortical tissue (*n* = 5; 2 male, 3 female; mean age 57.8 ± 6.7 years; mean eGFR 54.0 ± 11.0 ml/min/1.73m^2^) based on this histopathological assessment of the non-tumour kidney parenchyma. Kidney function (eGFR) was calculated using the CKD-EPI equation.

### Isolation and culture of human primary PTECs

Human primary PTECs were purified from kidney cortical tissue following the method of Glynne and Evans [17] and then cultured in Defined Medium (DM) as previously described [18]. Only human primary PTECs isolated from ‘non-fibrotic (control)’ cortical tissue were used for in vitro experiments (*n* = 9; Table S1). All PTECs used in experiments were at passage 4.

### Hypoxic and IL-1β treatment of human primary PTECs

PTECs were cultured to 70–80% confluence in DM in 6-well flat-bottom plates (unless otherwise specified). PTECs were then further cultured for 24–72 h in fresh DM (3 ml volume) for normoxic PTECs (21% O_2_) or, for hypoxic PTECs (1% O_2_), in hypoxia pre-conditioned fresh DM (3 ml volume) in an InvivO_2_ 1000 Hypoxia Workstation (Ruskinn, Laftec, Bayswater North, Victoria, Australia) in the absence or presence of 1 ng/ml recombinant human IL-1β (BioLegend, San Diego, CA, USA). PTECs were cultured for 24 h for RNA-sequencing (RNA-seq), 48 h for protein analysis (Western blotting and flow cytometry cell surface staining) or 72 h for cell cycle analysis (flow cytometry). PTECs were harvested using Buffer RLT (QIAGEN, Hilden, Germany) for downstream RNA-seq, urea lysis buffer (8 M urea, 1% SDS, 100 mM NaCl, 10 mM Tris (pH 7.5); all reagents from Sigma-Aldrich, St Louis, MO, USA) for Western blotting or by trypsin treatment for flow cytometry.

### Bulk RNA-seq, data processing and analysis

Total RNA was isolated using the RNeasy Mini kit (QIAGEN) according to the manufacturer’s instructions. RNA was isolated from four treatment groups (Normoxia Vehicle, Normoxia IL-1β, Hypoxia Vehicle, Hypoxia IL-1β) with four biological replicates (Patients 1, 2, 3, 4 in Table S1) per group (16 samples total). Detailed protocol for downstream RNA-seq, data processing and analysis is provided in Supplementary Methods.

### Protein expression and cell cycle analysis

PTECs were analyzed for protein expression/cell cycle distribution by Western blotting or flow cytometry (detailed protocols provided in Supplementary Methods).

### Cell proliferation measurements of human primary PTECs

Cell proliferation was investigated using the colorimetric MTT (3-(4,5-dimethylthiazol-2-yl)-2,5-diphenyltetrazolium bromide) Cell Proliferation Assay kit (Molecular Probes, Eugene, OR, USA) (detailed protocol provided in Supplementary Methods).

### Senescence associated β-galactosidase (SA-β-gal) staining of human primary PTECs

PTECs were seeded (100,000 cells/well in DM) onto sterile coverslips in 24-well flat-bottom plates to allow overnight adherence and then cultured for a further 72 h in fresh, conditioned DM under normoxic or hypoxic conditions in the absence or presence of 1 ng/ml recombinant human IL-1β. For inhibition of IL-1RI signalling, anti-human IL-1RI neutralising antibody (2.5 µg/ml; Goat polyclonal IgG; Cat. No. PA5-46930; Thermo Fisher Scientific, Waltham, MA, USA) or Goat IgG Isotype Control antibody (2.5 µg/ml; Cat. No. 31245; Thermo Fisher Scientific) were added to PTEC cultures for the 72 h treatment period. In selected ‘senolytic’ experiments, following the 72 h treatment period, cells were further cultured in fresh DM containing 10 µM quercetin and 5 nM dasatinib (both from Sigma-Aldrich) or 0.01% dimethyl sulfoxide (DMSO; Sigma-Aldrich) vehicle control for an additional 24 h for SA-β-gal staining or 48 h for cytokine analysis of culture supernatants using the LEGENDplex™ Human Essential Immune Response Panel multiplex bead array (BioLegend). SA-β-gal activity was determined using the Senescence β-Galactosidase Staining Kit (Cell Signaling Technology, Danvers, MA, USA) following the manufacturer’s instructions (detailed protocol provided in Supplementary Methods).

### IF staining of kidney tissue

Remaining ‘non-fibrotic (control)’ (*n* = 4; 3 male, 1 female; mean age 54.2 ± 18.4 years; mean eGFR 80.8 ± 10.9 ml/min/1.73m^2^) and ‘fibrotic’ cortical tissue samples (*n* = 5; 2 male, 3 female; mean age 57.8 ± 6.7 years; mean eGFR 54.0 ± 11.0 ml/min/1.73m^2^) were used for in situ IF staining studies.

For IL-1RI profiling, frozen 7 µm tissue sections were fixed with 75% acetone: 25% ethanol for 5 min at room temperature, followed by a protein block with 10% Donkey Serum for 30 min at room temperature. Sections were subsequently probed with primary antibodies against IL-1RI (1:70; Goat polyclonal IgG; Cat. No. AF269SP; R&D Systems, Minneapolis, MN, USA) and PTEC marker aquaporin-1 (AQP-1; 1:50; Mouse monoclonal IgG; Clone B-11; Cat. No. sc-25287; Santa Cruz, Dallas, TX, USA) overnight at 4 °C in a humidified chamber. Fluorescent detection was obtained by secondary incubation with Alexa Fluor™ Plus 647 anti-mouse IgG and Alexa Fluor™ Plus 555 anti-goat IgG (1:500 dilutions; both from Thermo Fisher) for 30 min at room temperature.

For in situ SA-β-gal profiling, frozen 7 µm tissue sections were fixed with 4% paraformaldehyde (PFA) in PBS for 20 min at room temperature, washed thrice with PBS, stained with 2 µM Spider-β-gal (Dojindo, Rockville, MD, USA) in McIlvaine buffer (0.1 M citric acid and 0.2 M sodium phosphate; pH 6.0) for 1 h at 37 °C in a humidified chamber, washed thrice with PBS, followed by a protein block with 10% Donkey Serum for 30 min at room temperature. Sections were then probed with an AQP-1 primary antibody (1:100; Rabbit polyclonal IgG; Cat. No. sc-20810; Santa Cruz) for 1 h at room temperature, followed by secondary antibody incubation with Alexa Fluor™ Plus 647 anti-rabbit IgG (1:500; Thermo Fisher) for 30 min at room temperature.

For both IL-1RI and SA-β-gal staining protocols, nuclei were subsequently stained with DAPI (1:10,000; Sigma-Aldrich), with slides coverslipped in fluorescence mounting medium (Agilent Technologies, Santa Clara, CA, USA) and visualized using a Zeiss 780 NLO confocal microscope (Carl Zeiss, Hamburg, Germany). Quantitative image analysis of kidney tissue was performed using QuPath (version 0.3.2) [19]. Briefly, IL-1RI^+^ cells, SA-β-gal^+^ cells and AQP-1^+^ cells were enumerated in four randomly selected areas for each tissue section. Each area was assessed using the Watershed Cell Detection algorithm [19, 20], with staining intensity threshold-based cell classification identifying positive cells for quantitative analysis.

### Flow cytometric analysis of urinary PTECs

Urine samples were obtained with informed written consent from additional patients undergoing diagnostic kidney biopsy (*n* = 39; 19 male, 20 female; Table S2), with study approval from the Royal Brisbane and Women’s Hospital Human Research Ethics Committee (2002/011 and 2006/072). Urine samples (50-100 ml) were collected 1-2 h prior to the biopsy procedure and stratified into two cohorts based on the histopathological absence (<5%) or presence (≥5%) of interstitial fibrosis in the contemporaneous kidney biopsy. According to this histopathological criterion [16], urine specimens were grouped into ‘control/non-fibrotic’ (*n* = 7; 3 males, 4 females; mean age 44.4 ± 18.3 years; mean eGFR 61.7 ± 36.4 ml/min/1.73m^2^) or ‘fibrotic’ urine specimens (*n* = 32; 16 males, 16 females; mean age 54.8 ± 17.4 years; mean eGFR 41.3 ± 23.2 ml/min/1.73m^2^) (Table S2).

Fresh urine samples were immediately centrifuged at 650xg for 10 min at 4 °C to pellet urinary cells. Urinary supernatant was also retained and available from selected samples for downstream urinary IL-1β detection (i.e., patients without an asterisk in Table S2). For these selected samples, 50 ml aliquots of urinary supernatant were mixed with one tablet of protease inhibitor cocktail dissolved in 2 ml H_2_O (Roche Diagnostics, Mannheim, Germany) and stored at −80 °C until examined for urinary IL-1β levels using the Human IL-1β ELISA MAX™ Deluxe kit (Biolegend) following the manufacturer’s recommendations.

Urinary cells were initially stained with LIVE/DEAD^®^ Fixable Near-IR Dead Cell Stain Kit (Life Technologies, Grand Island, NY, USA) to exclude non-viable cells, then incubated with Human TruStain FcX™ Blocking Solution (Biolegend) at room temperature for 5–10 min and stained on ice for 30 min with combinations of test (Table S3) or isotype-matched control antibodies in cold FACS buffer. Cells were then fixed with 1% PFA for 10 min at room temperature, washed twice with FACS buffer and labelled with 1 µM Spider-β-gal (Dojindo) in McIlvaine buffer (pH 6.0) or buffer alone for 30 min at 37 °C.

Flow-Count Fluorospheres™ (Beckman Coulter, Brea, CA, USA) were used for direct determination of absolute counts following the manufacturer’s recommendations. Briefly, target cell concentrations (cells/µl) were calculated as: total number of target cells counted divided by total number of Fluorospheres counted multiplied by Flow-Count Fluorospheres™ assayed concentration. This value was then multiplied by the total sample volume to obtain absolute counts for each target cell population. Absolute cell counts were then normalised to cell numbers per ml of urine. Cell acquisition was performed on an LSR Fortessa (BD Biosciences, San Jose, CA, USA) and data analyzed with FlowJo software (TreeStar, Ashland, OR, USA).

### Statistics

Sample sizes were selected based on previous publications from our laboratory with similar experimental design [21]. Data were normalised as a fold change relative to control conditions (i.e. normoxia+vehicle) for each donor. Statistical tests for in vitro phenotyping (flow cytometry, Western blotting) and functional assays, in situ IF profiling and ex vivo urinary PTEC studies were performed using GraphPad Prism (version 9.0.1; GraphPad Software, La Jolla, CA, USA). Multiple paired comparisons were performed using a one-way analysis of variance (ANOVA) with Tukey’s multiple-comparison post-hoc test. Statistical comparisons between unpaired groups were performed using a Welch’s t-test. Absolute urinary PTEC numbers were correlated with levels of interstitial fibrosis in contemporaneous kidney biopsies, patient eGFR and urinary IL-1β levels by Spearman correlation analysis. *P*-values ≤ 0.05 were considered statistically significant.

## Results

### Human primary proximal tubular epithelial cells (PTECs) up-regulate IL-1RI under hypoxic-inflammatory conditions

**Fig. 1:**
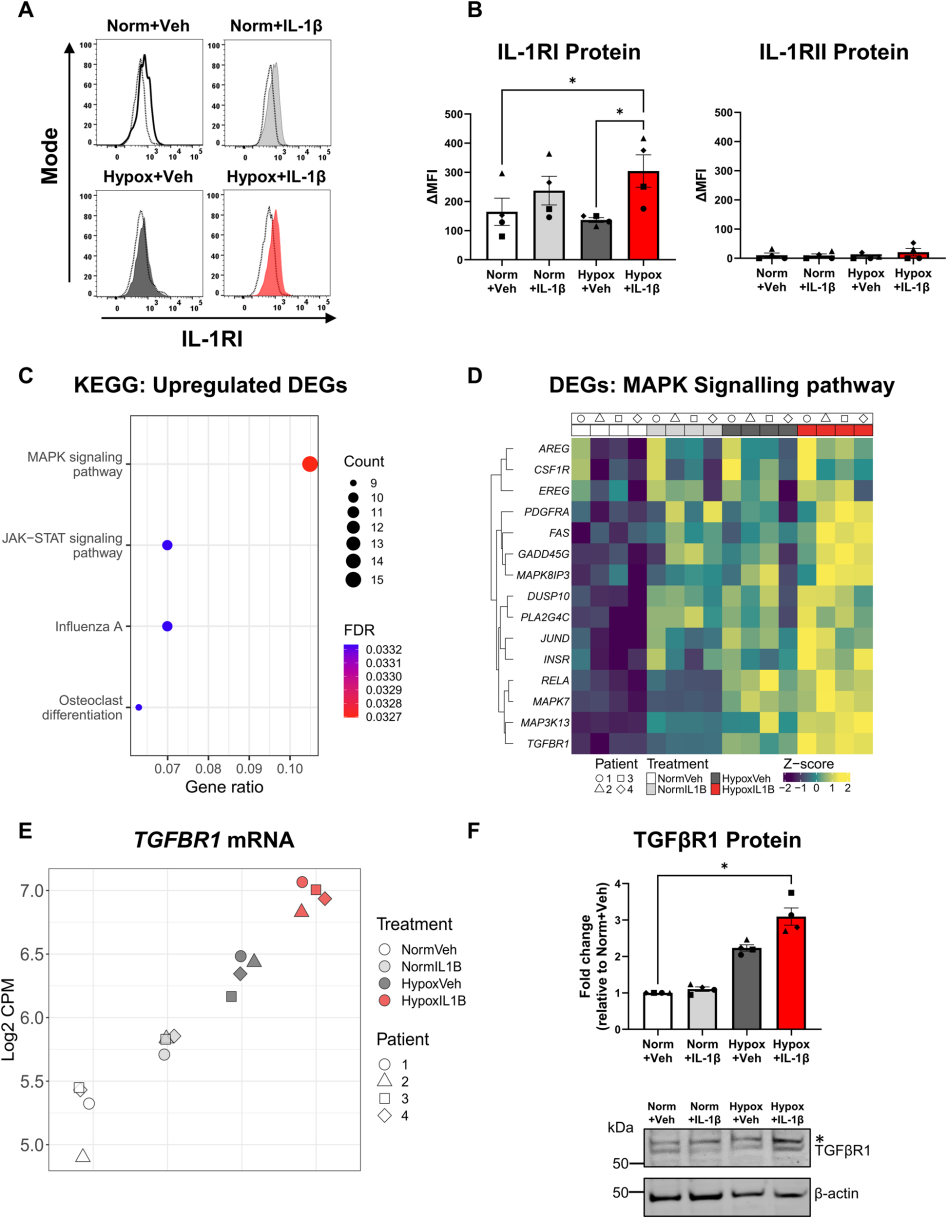
Hypoxia and IL-1β synergistically up-regulate IL-1RI surface expression and mitogen-activated protein kinase (MAPK) signalling pathways in human primary proximal tubular epithelial cells (PTECs). **A** IL-1RI surface staining compared with isotype control (dashed line) of human primary PTECs following 48 h culture under normoxic (norm) or hypoxic (hypox) conditions in the absence (vehicle; veh) or presence of IL-1β (gated on live, singlet cells). Representative histograms from one of four donor PTEC are presented. **B** IL-1RI (left panel) and IL-1RII (right panel) protein expression levels (measured as delta median fluorescence intensity (ΔMFI), representing MFI test minus MFI isotype control) for PTECs cultured under normoxic (norm) or hypoxic (hypox) conditions in the absence (vehicle; veh) or presence of IL-1β. Bar graphs represent mean ± SEM. Symbols represent individual donor PTECs; *n* = 4. *P < 0.05, one-way ANOVA with Tukey’s multiple-comparison test. **C** Significantly (False Discovery Rate [FDR] <0.05) enriched KEGG pathways identified from ‘unique’ up-regulated differentially expressed genes (DEGs) for the Hypoxia+IL-1β vs. Normoxia+Vehicle contrast. The count (represented by point size) is the number of DEGs annotated in the enriched KEGG pathway. The gene ratio (x-axis) is the count divided by the total number of DEGs with annotations across the collection of KEGG pathways. FDR is represented by point colour. **D** Heatmap showing normalised expression values (scaled and centred) for unique DEGs from the Hypoxia+IL-1β vs. Normoxia+Vehicle contrast contributing to the KEGG MAPK signalling pathway. Expression values for all samples are shown, with samples (columns) ordered by treatment then patient. **E** Scatterplot of normalised gene expression values (log_2_ counts per million [CPM]) for *TGFBR1* across all PTEC samples. **F** Top panel: Fold changes (relative to Normoxia+Vehicle) in TGFβR1 protein levels (as a ratio of loading control β-actin) for PTECs cultured for 48 h under normoxic (norm) or hypoxic (hypox) conditions in the absence (vehicle; veh) or presence of IL-1β. Bar graphs represent mean ± SEM. Symbols represent individual donor PTECs; *n* = 4. *P < 0.05, one-way ANOVA with Tukey’s multiple-comparison test. Bottom panel: TGFβR1 Western blot for PTECs cultured under normoxic (norm) or hypoxic (hypox) conditions in the absence (vehicle; veh) or presence of IL-1β (20 µg total protein per lane). Representative images from one of four donor PTECs are presented. The upper, non-specific band in the TGF-βR1 blot is indicated with an asterisk (*) and was excluded from densitometry analysis. Full and uncropped Western blot available as Supplementary Material.

Hypoxia and inflammatory IL-1β signalling are independent drivers of tubulointerstitial fibrosis, the histological hallmark of CKD [12, 13]. Here, we examined the pathobiological mechanisms by which hypoxia and IL-1β synergistically mediate maladaptive PTEC repair in CKD. Ex vivo patient-derived PTECs were cultured under normoxic (21% O_2_, “Norm”) or hypoxic (1% O_2_; “Hypox”) conditions in the absence (vehicle, “Veh”) or presence of IL-1β. Hypoxic PTECs cultured with IL-1β displayed significantly increased expression of IL-1RI (CD121a) protein compared with PTECs cultured under normoxia or hypoxia alone (Fig. 1A, B). No to minimal expression of decoy/inhibitory receptor, IL-1RII (CD121b), was detected across all treatment conditions (Fig. 1B). These results indicate that hypoxia in combination with IL-1β are required to up-regulate IL-1RI expression and augment PTEC sensitivity to additional IL-1β stimulation.

### Human primary PTECs display a discrete transcriptomic profile with up-regulated mitogen-activated protein kinase (MAPK) signalling under hypoxic-inflammatory conditions

To interrogate the transcriptome of PTECs in hypoxic-inflammatory conditions, we performed bulk RNA-seq of four individual PTEC donor experiments across the four treatment groups. Selection of protein-coding genes and removal of low abundance transcripts resulted in 14,362 genes for downstream analysis. Of these genes, established PTEC markers, *ANPEP* (CD13), *HAVCR1* (KIM-1) and *MME* (CD10) [22], were expressed across all conditions (Fig. S1A), confirming the PTEC identity of primary cells. Principal component analysis (PCA) revealed that PC1 largely separated the samples according to oxygen conditions (Fig. S1B). Along PC2 we identified a strong donor effect (Fig. S1B, top panel), while PC1 vs. PC3 showed clear separation of the samples by treatment group (Fig. S1B, bottom panel). Contrasts were performed to identify differentially expressed genes (DEGs; false discovery rate (FDR) < 0.05) between conditions (Table S4), with 2041 DEGs (1364 up-regulated, 677 down-regulated) identified between Hypoxia+IL-1β-treated vs. Normoxia+Vehicle PTECs (Fig. S1C, Tables S5–S9). Of these 2041 DEGs, 692 genes (347 up-regulated, 345 down-regulated) were ‘unique’ to the Hypoxia+IL-1β vs. Normoxia+Vehicle contrast (Fig. S1D, Table S10) and retained for downstream pathway enrichment analysis.

The functional associations of the 347 ‘unique’ up-regulated DEGs were examined by overrepresentation analysis using the Kyoto Encyclopaedia of Genes and Genomes (KEGG) database. Four significant KEGG pathways (FDR < 0.05) were identified: (i) the mitogen-activated protein kinase (MAPK) signalling pathway; (ii) Janus kinase (JAK)-signal transducer and activator of transcription (STAT) signalling pathway; (iii) Influenza A; and (iv) osteoclast differentiation (Fig. 1C, Table S11). Of the 15 MAPK signalling-associated DEGs (Fig. 1D), transforming growth factor beta receptor 1 (*TGFBR1*) was one of the most abundant genes in the Hypoxia+IL-1β samples (highest log_2_ counts per million (CPM) value; Fig. 1E). RNA-seq data was supported by Western blot analysis, with TGFβR1 protein significantly increased in Hypoxia+IL-1β-treated PTECs compared with Normoxia+Vehicle PTECs (Fig. 1F). These TGFβR1 expression data suggest that PTEC responsiveness to pro-fibrotic TGF-β1 signalling will be elevated under hypoxic-inflammatory conditions.

### Human primary PTEC display a transcriptomic profile associated with down-regulated cell cycling under hypoxic-inflammatory conditions

**Fig. 2:**
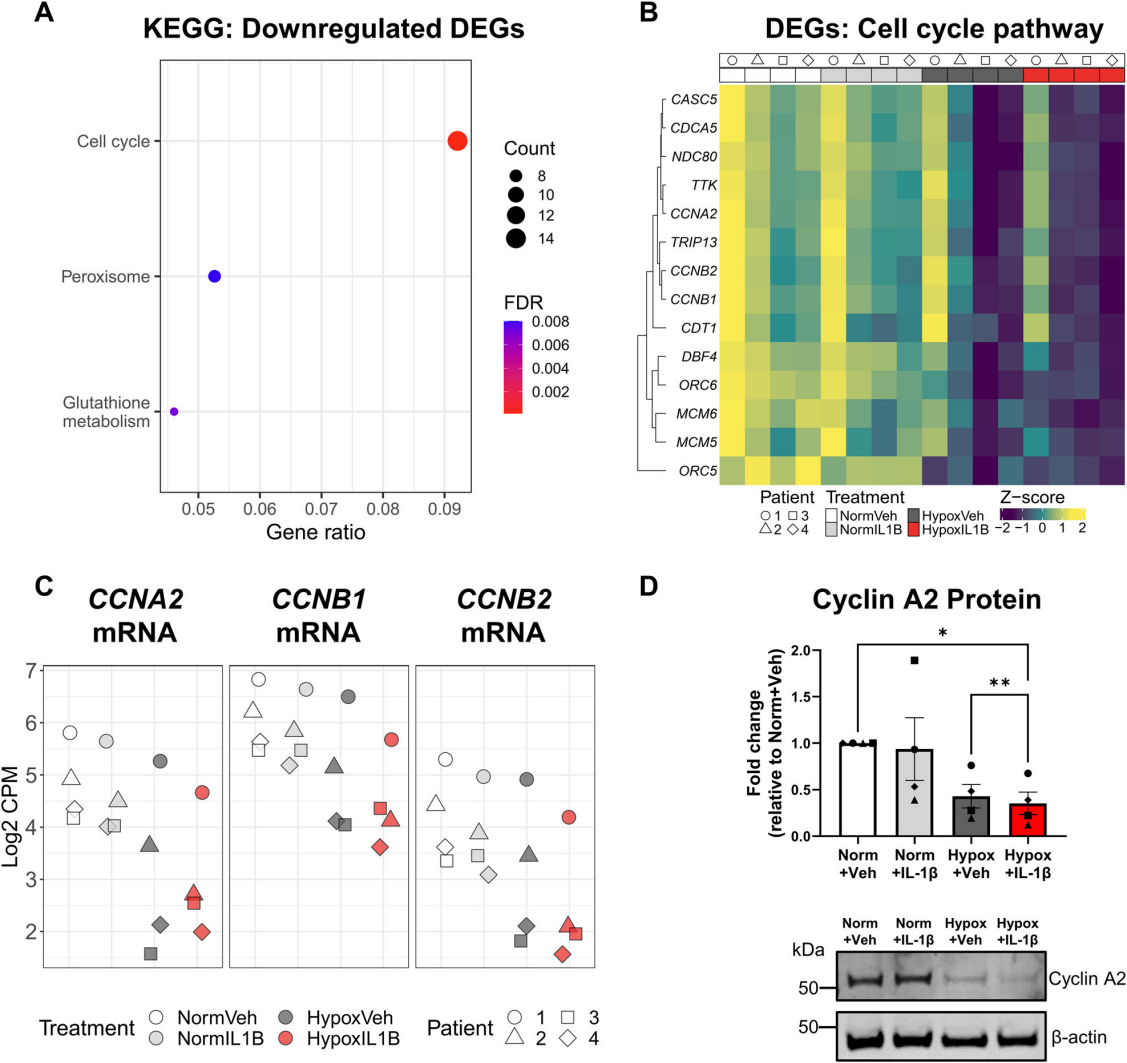
Hypoxia and IL-1β synergistically down-regulate cell cycling pathways in human primary PTECs. **A** Significantly (FDR < 0.05) enriched KEGG pathways identified from ‘unique’ down-regulated differentially expressed genes (DEGs) for the Hypoxia+IL-1β vs. Normoxia+Vehicle contrast. The count (represented by point size) is the number of DEGs annotated in the enriched KEGG pathway. The gene ratio (x-axis) is the count divided by the total number of DEGs with annotations across the collection of KEGG pathways. FDR is represented by point colour. **B** Heatmap showing normalised gene expression values (scaled and centred) for unique DEGs from the Hypoxia+IL-1β vs. Normoxia+Vehicle contrast contributing to the KEGG Cell cycle pathway. Expression values for all samples are shown, with samples (columns) ordered by treatment then patient. **C** Scatterplot of normalised expression values (log_2_ CPM) for *CCNA2*, *CCNB1* and *CCNB2* across all PTEC samples. **D** Top panel: Fold changes (relative to Normoxia+Vehicle) in Cyclin A2 protein levels (as a ratio of loading control β-actin) for PTECs cultured for 48 h under normoxic (norm) or hypoxic (hypox) conditions in the absence (vehicle; veh) or presence of IL-1β. Bar graphs represent mean ± SEM. Symbols represent individual donor PTECs; *n* = 4. *P < 0.05, **P < 0.01, one-way ANOVA with Tukey’s multiple-comparison test. Bottom panel: Cyclin A2 Western blot for PTECs cultured under normoxic (norm) or hypoxic (hypox) conditions in the absence (vehicle; veh) or presence of IL-1β (20 µg total protein per lane). Representative images from one of four donor PTECs are presented. Full and uncropped Western blot available as Supplementary Material.

Equivalent analysis of the 345 ‘unique’ down-regulated DEGs in the Hypoxia+IL-1β vs. Normoxia+Vehicle contrast identified three significant KEGG pathways: (i) cell cycle pathway, with the highest gene ratio; (ii) peroxisome; and (iii) glutathione metabolism (Fig. 2A, Table S12). Of the 14 ‘unique’ down-regulated DEGs in the cell cycle pathway were cyclins (*CCNA2*, *CCNB1*, *CCNB2*; Fig. 2B, C), central mediators of cell cycle progression with previous reports of reduced expression in senescent cells [23]. Cyclin A2 protein expression (encoded by *CCNA2*) was also shown to be significantly decreased in Hypoxia+IL-1β-treated PTECs compared with PTECs cultured under normoxia or hypoxia alone (Fig. 2D). Further examination of the ‘unique’ DEGs in the Hypoxia+IL-1β vs. Normoxia+Vehicle contrast also identified reduced expression of lamin B1 (*LMNB1*) (Table S10), an established hallmark of nuclear envelope disruption and cellular senescence [24].

### PTEC senescence under hypoxic-inflammatory conditions is mediated via IL-1RI signalling

**Fig. 3:**
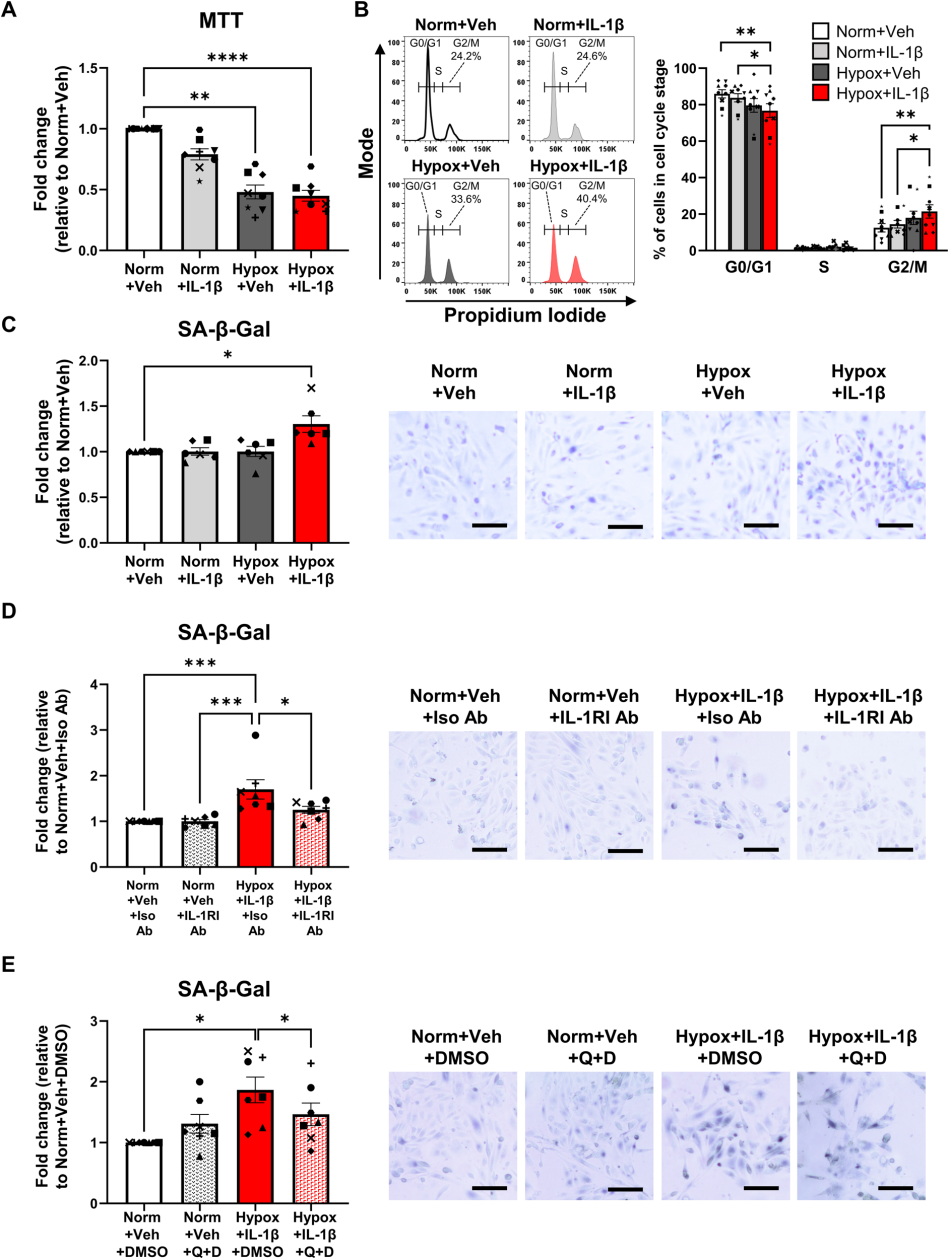
Hypoxia in synergy with IL-1β triggering of IL-1RI mediate G2/M arrest and senescence of human primary PTECs. **A** Fold changes (relative to Normoxia+Vehicle) in cell proliferation (MTT assay) for PTECs cultured for 72 h under normoxic (norm) or hypoxic (hypox) conditions in the absence (vehicle; veh) or presence of IL-1β. Bar graphs represent mean ± SEM. Symbols represent individual donor PTECs; *n* = 8. **P < 0.01, ****P < 0.0001, one-way ANOVA with Tukey’s multiple-comparison test. **B** Left panel: Representative cell cycle analysis for PTECs cultured for 72 h under normoxic (norm) or hypoxic (hypox) conditions in the absence (vehicle; veh) or presence of IL-1β. The percentage of cells in G2/M phase arrest for each histogram are presented. Representative gating strategy showing exclusion of debris/dead cells and aggregates/doublets prior to cell cycle analysis is outlined in Fig. S2C. Right panel: Cell cycle analysis (% of cells in each cell cycle stage) for PTECs under different culture conditions. Bar graphs represent mean ± SEM. Symbols represent individual donor PTECs; *n* = 9. *P < 0.05, **P < 0.01, one-way ANOVA with Tukey’s multiple-comparison test. **C** Left panel: Fold changes (relative to Normoxia+Vehicle) in senescence associated β-galactosidase (SA-β-gal) activity (measured as % SA-β-gal^+^ cells) for PTECs cultured for 72 h under normoxic (norm) or hypoxic (hypox) conditions in the absence (vehicle; veh) or presence of IL-1β. Bar graphs represent mean ± SEM. Symbols represent individual donor PTECs; *n* = 6. *P < 0.05, one-way ANOVA with Tukey’s multiple-comparison test. Right panel: Representative SA-β-gal staining for PTECs cultured under normoxic (norm) or hypoxic (hypox) conditions in the absence (vehicle; veh) or presence of IL-1β. Scale bars represent 100 µm. **D** Left panel: Fold changes (relative to Normoxia+Vehicle+Isotype Ab) in SA-β-gal activity (measured as % SA-β-gal^+^ cells) for PTECs cultured for 72 h under normoxic conditions alone (Norm+Veh) or hypoxic conditions with IL-1β (Hypox+IL-1β) in the presence of isotype control antibody (Iso Ab) or neutralising IL-1RI Ab. Bar graphs represent mean ± SEM. Symbols represent individual donor PTECs; *n* = 7. *P < 0.05, ***P < 0.001, one-way ANOVA with Tukey’s multiple-comparison test. Right panel: Representative SA-β-gal staining; scale bars represent 100 µm. **E** Left panel: Fold changes (relative to Normoxia+Vehicle+DMSO) in SA-β-gal activity (measured as % SA-β-gal^+^ cells) for PTECs initially cultured for 72 h under normoxic conditions alone (Norm+Veh) or hypoxic conditions in the presence of IL-1β (Hypox+IL-1β), followed by additional 24 h treatment in fresh DM without (DMSO vehicle control) or with quercetin+dasatinib (Q + D). Bar graphs represent mean ± SEM. Symbols represent individual donor PTECs; *n* = 7. *P < 0.05, one-way ANOVA with Tukey’s multiple-comparison test. Right panel: Representative SA-β-gal staining; scale bars represent 100 µm.

Mechanistic investigations were performed to investigate cell cycle arrest/senescence in Hypoxia+IL-1β-treated PTECs. Hypoxic PTECs cultured alone and in the presence of IL-1β displayed significantly reduced cell proliferation (MTT assay) compared with normoxic PTECs, with highest significance observed in Hypoxia+IL-1β-treated PTECs (*P*-value < 0.0001; Fig. 3A). We examined the contribution of hypoxic cell death to this reduced MTT activity, showing significantly increased cell death (percentage of Near-IR viability dye^+^ cells) only in PTECs cultured under hypoxia alone compared to both Normoxia+Vehicle PTECs and Hypoxia+IL-1β-treated PTECs (Fig. S2A). These data suggest that the reduced MTT activity in Hypoxia+Vehicle PTECs is due to decreased viability, but cell cycle arrest in Hypoxia+IL-1β-treated PTECs. Supporting this concept, gene set enrichment analysis (GSEA) for the Hypoxia+IL-1β vs. Hypoxia+Vehicle (bulk-RNA seq) contrast using the gene sets “Reactome Cellular Senescence” and “Reactome senescence associated secretory phenotype SASP” revealed both ‘senescent’ gene sets to be significantly enriched in the Hypoxia+IL-1β condition (FDR < 0.05, Fig. S2B).

Further in vitro studies showed an increased percentage of PTECs arrested in the G2/M cell cycle phase following combined treatment with Hypoxia+IL-1β (Fig. 3B and Fig. S2C for gating strategy). We also observed significantly elevated expression of senescence marker p21 (Fig. S2D) and senescence associated β-galactosidase (SA-β-gal) activity (X-gal staining; Fig. 3C) restricted to Hypoxia+IL-1β-treated PTECs compared with normoxic PTECs. The addition of an IL-1RI neutralising antibody during the treatment period significantly attenuated SA-β-gal activity in hypoxic PTECs cultured with IL-1β (Fig. 3D), whilst additional culture in fresh DM with quercetin plus dasatinib (Q + D), the established senolytic combination to clear senescence [25], also reduced senescent cell burden in Hypoxia+IL-1β-treated PTECs (Fig. 3E).

Screening for senescence-associated secretory phenotype (SASP) factors in culture supernatants identified significantly increased TGF-β1, monocyte chemoattractant protein (MCP)-1 and IL-6 levels in Hypoxia+IL-1β-treated PTECs compared with normoxic PTECs (Fig. S3A–C). A similar profile was also observed in normoxic PTECs cultured with IL-1β (Fig. S3A–C). There were no significant changes in IL-8 and tumour necrosis factor (TNF)-α expression levels between PTEC culture conditions (Fig. S3D-E). Of note, in ‘senolytic’ experiments, further culture in fresh DM with quercetin plus dasatinib (Q + D) significantly attenuated IL-6 production in only Hypoxia+IL-1β-treated PTECs (Fig. S3F), with other lower-abundance cytokines not detectable in culture supernatants (data not shown). These collective data support a complex mechanism whereby hypoxia in synergy with IL-1β triggering of IL-1RI mediate human PTEC senescence via G2/M phase arrest and downstream production of specialized SASP factors (e.g. IL-6).

### Significantly elevated PTEC IL-1RI expression and senescence in fibrotic kidneys

**Fig. 4:**
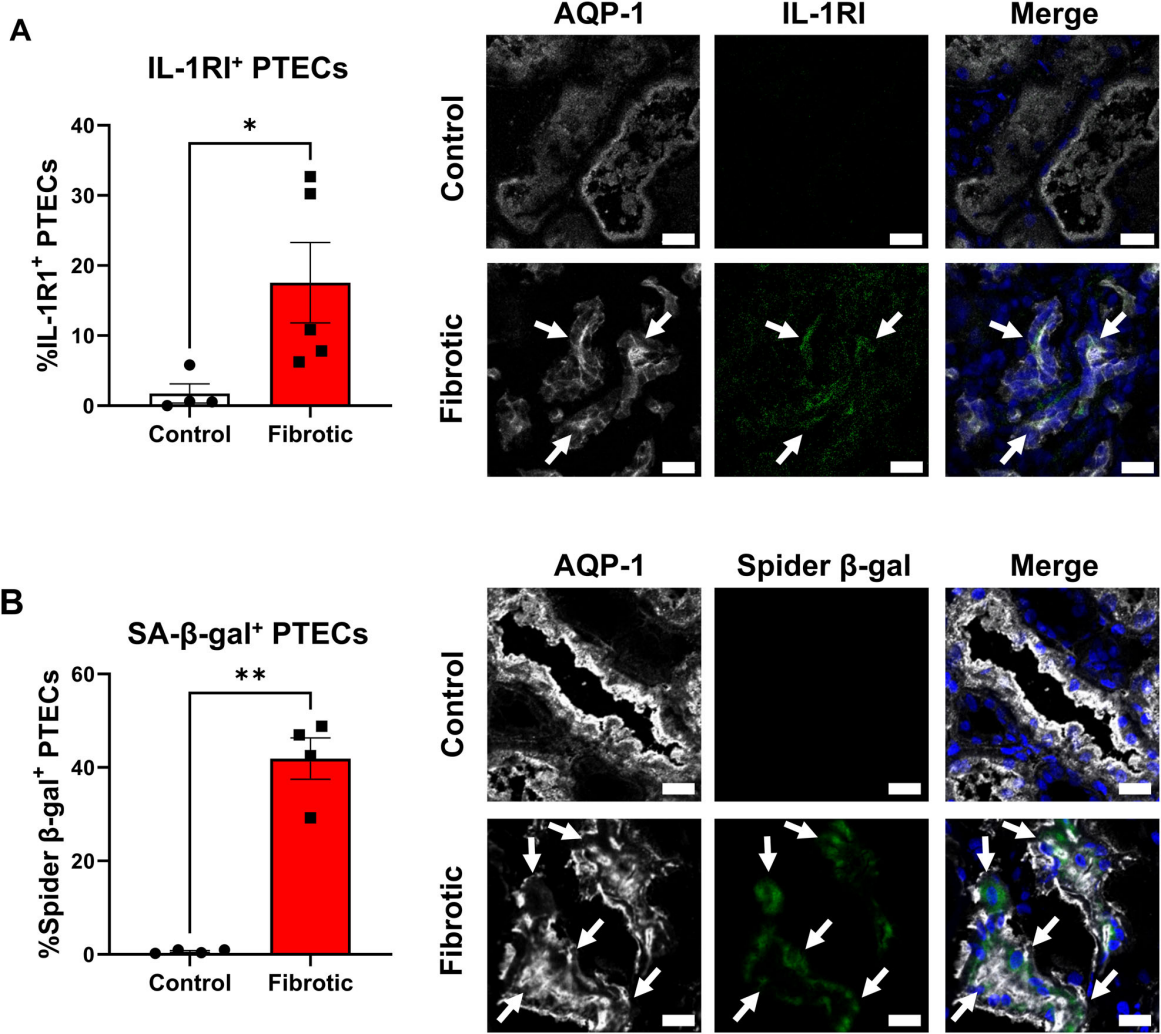
Significantly elevated PTEC IL-1RI expression and senescence in fibrotic kidneys. **A** Left panel: Quantitative analysis of % IL-1RI^+^ PTECs (proportion of AQP-1^+^ cells (i.e., PTECs) expressing IL-1RI) in control (non-fibrotic; *n* = 4) and fibrotic kidney tissue (*n* = 5). Points represent values for individual donor tissue samples. Results represent mean ± SEM of values from four randomly selected areas for each tissue sample. *P < 0.05, Welch’s *t*-test. Right panel: Representative immunofluorescent labelling of control (non-fibrotic) and fibrotic kidney tissue stained for PTEC marker aquaporin-1 (AQP-1; white), IL-1RI (green) and DAPI (blue). Scale bars represent 20 µm. IL-1RI^+^ PTECs are highlighted with white arrows. **B** Left panel: Quantitative analysis of % SA-β-gal^+^ PTECs (proportion of AQP-1^+^ cells (i^.^e., PTEC) with positive Spider-β-gal staining) in control (non-fibrotic; *n* = 4) and fibrotic kidney tissue (*n* = 4). Points represent values for individual donor tissue samples. Results represent mean ± SEM of values from four randomly selected areas for each tissue sample. **P < 0.01, Welch’s *t*-test. Right panel: Representative immunofluorescent images of control (non-fibrotic) and fibrotic kidney tissue labelled with PTEC marker AQP-1 (white), Spider-β-gal (green) and DAPI (blue). Scale bars represent 20 µm. SA-β-gal^+^ PTECs are highlighted with white arrows. Lower magnification immunofluorescent images are presented in Fig. S4.

To examine the biological significance of senescence in Hypoxia+IL-1β-treated PTECs, we extended our analysis to in situ profiling of PTECs in the hypoxic-inflammatory microenvironment of human fibrotic kidneys. PTECs were identified as aquaporin-1 (AQP-1)^+^ tubular cells [26]. IF staining of fibrotic kidney tissue revealed PTEC expression of IL-1RI (Fig. 4A) and, for the first time using the fluorescent Spider-β-gal probe, PTEC SA-β-gal activity (Fig. 4B and Fig. S4). Quantitative analysis showed significantly increased proportions of IL-1RI^+^ and SA-β-gal^+^ PTECs in fibrotic kidneys compared with control (non-fibrotic) tissue (Fig. 4A, B). These results associate PTEC IL-1RI expression and senescence with the development of tubulointerstitial fibrosis.

### Significantly elevated numbers of senescent urinary PTECs in patients with tubulointerstitial fibrosis

**Fig. 5:**
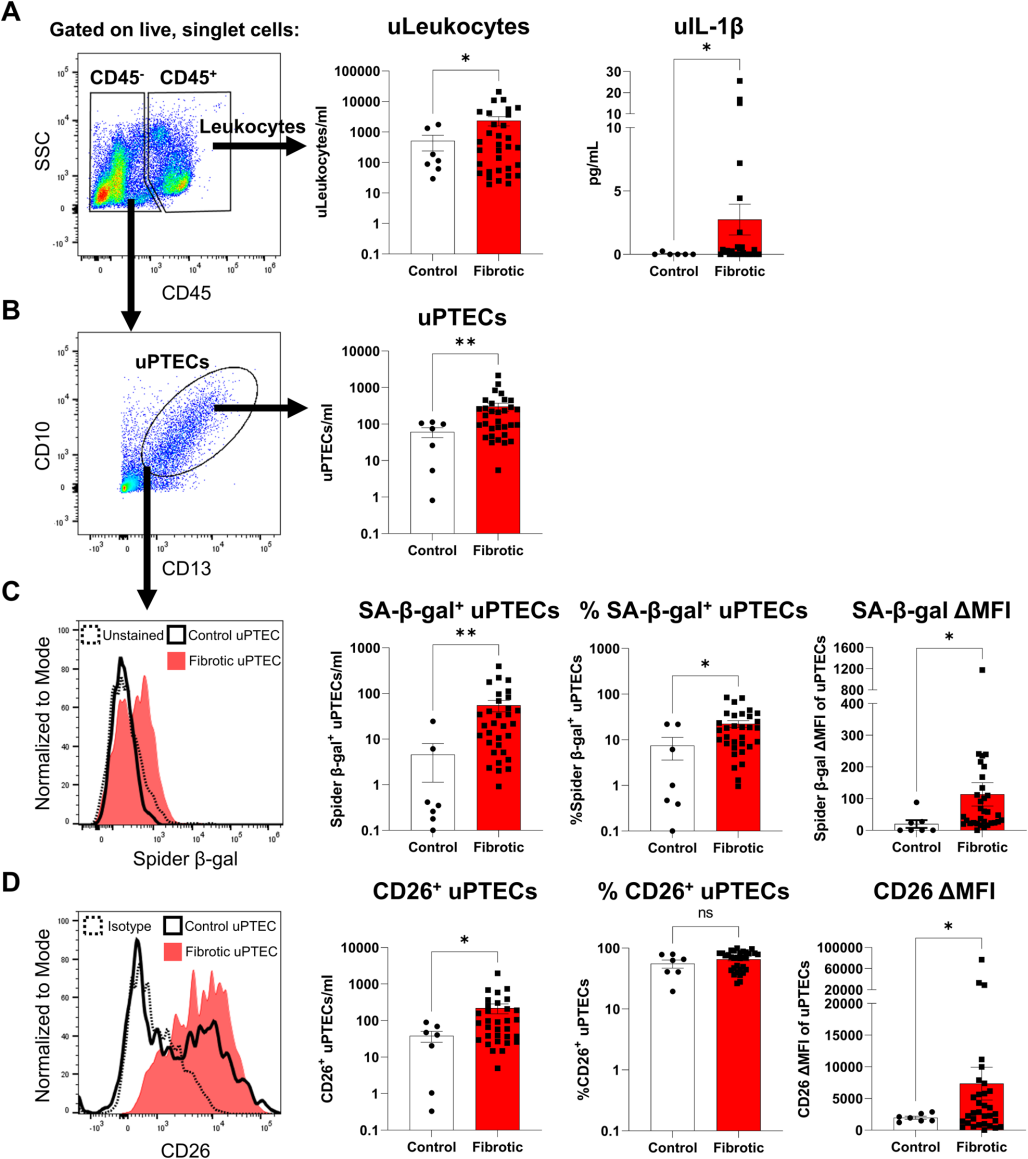
Significantly elevated numbers and proportions of senescent urinary PTECs in patients with tubulointerstitial fibrosis. **A** Left panel: Flow cytometric gating strategy (gated on live, singlet cells) used to identify CD45^-^ cells and CD45^+^ leukocytes in urine samples collected at the time of kidney biopsy. Representative image from one of 32 urine samples from an individual with tubulointerstitial fibrosis. SSC: side scatter. Middle panel: Absolute numbers of urinary leukocytes (uLeukocytes/ml urine) from kidney biopsy patients stratified based on the histological absence (control/non-fibrotic; *n* = 7) or presence of interstitial fibrosis (*n* = 32). Points represent values for individual urine samples. Bar graphs represent mean ± SEM. *P < 0.05, Welch’s t-test. Right panel: Levels of urinary IL-1β (uIL-1β; pg/ml urine) from kidney biopsy patients stratified based on the histological absence (control; *n* = 6) or presence of interstitial fibrosis (*n* = 27). Points represent values for individual urine samples. Bar graphs represent mean ± SEM. *P < 0.05, Welch’s *t*-test. **B** Left panel: Identification of urinary PTECs (uPTECs; CD13^+^ CD10^+^ cells within the CD45^-^ fraction). Right panel: Absolute numbers of urinary PTECs (uPTECs/ml urine) from kidney biopsy patients stratified based on the histological absence (control; *n* = 7) or presence of interstitial fibrosis (*n* = 32). Points represent values for individual urine samples. Bar graphs represent mean ± SEM. **P < 0.01, Welch’s *t*-test. **C** Left panel: Relative SA-β-gal activity (Spider-β-gal staining) of uPTECs from kidney biopsy patients without (control uPTEC; black unfilled) and with interstitial fibrosis (fibrotic uPTEC; red filled) compared to unstained control (dashed line). Right panels: Quantitative analysis of absolute numbers of SA-β-gal^+^ uPTECs (SA-β-gal^+^ uPTECs/ml urine), % SA-β-gal^+^ uPTECs (proportion of CD13^+^ CD10^+^ cells with positive Spider^-^β-gal staining) and Spider-β-gal fluorescence intensity of uPTECs (measured as delta median fluorescence intensity (ΔMFI), representing MFI test minus MFI unstained control) from kidney biopsy patients stratified based on the histological absence (control; *n* = 7) or presence of interstitial fibrosis (*n* = 32). Points represent values for individual urine samples. Bar graphs represent mean ± SEM. *P < 0.05, **P < 0.01, Welch’s *t*-test. **D** Left panel: Relative expression of CD26 on uPTECs from kidney biopsy patients without (control uPTEC; black unfilled) and with interstitial fibrosis (fibrotic uPTEC; red filled) compared to isotype control (dashed line). Right panels: Quantitative analysis of absolute numbers of CD26^+^ uPTECs (CD26^+^ uPTECs/ml urine), % CD26^+^ uPTECs (proportion of CD13^+^ CD10^+^ cells expressing CD26) and CD26 fluorescence intensity of uPTECs (measured as ΔMFI, representing MFI test minus MFI isotype control) from kidney biopsy patients stratified based on the histological absence (control; *n* = 7) or presence of interstitial fibrosis (*n* = 32). Points represent values for individual urine samples. Bar graphs represent mean ± SEM. *P < 0.05, Welch’s *t*-test (ns – not significant).

We next examined urinary PTECs as an ex vivo representation of the tubulointerstitial microenvironment. Urine samples were collected from patients undergoing diagnostic kidney biopsy and stratified into two cohorts based on the histopathological absence (<5%; *n* = 7) or presence (≥5%; *n* = 32) of interstitial fibrosis in the contemporaneous kidney biopsy (Table S2). Urinary cells were isolated from ‘control (non-fibrotic)’ vs. ‘fibrotic’ urine specimens and labelled for multi-parameter flow cytometry. We used an established gating strategy to identify discrete urinary leukocyte (uLeukocytes; CD45^+^ cells; Fig. 5A – left panel) and urinary PTEC (uPTECs; CD45^-^ CD13^+^ CD10^+^ cells; Fig. 5B - left panel) populations [22]. Quantitative analysis (cells per ml urine) showed significantly elevated numbers of uLeukocytes and uPTECs in ‘fibrotic’ urine compared with ‘control’ urine (Fig. 5A, B; middle panels). Notably, levels of urinary IL-1β were also significantly increased in ‘fibrotic’ urine (Fig. 5A; right panel), suggestive of a functional role for inflammatory IL-1β signalling in tubulointerstitial fibrosis.

Senescent phenotyping of uPTECs (Spider-β-gal staining) showed significantly elevated numbers and proportions of SA-β-gal^+^ uPTECs in ‘fibrotic’ urine compared with ‘control’ urine, with Spider-β-gal fluorescence intensity of uPTECs in ‘fibrotic’ urine also elevated (Fig. 5C). Similar increases in absolute numbers and fluorescence intensity of uPTECs in ‘fibrotic’ urine were observed with senescent marker CD26 (dipeptidyl-peptidase 4; DPP4) [27, 28] (Fig. 5D). We also report significant positive correlations between the degree of interstitial fibrosis and both SA-β-gal^+^ uPTEC numbers (*r* = 0.3792, *p* = 0.0173) and Spider-β-gal fluorescence intensity of uPTECs (*r* = 0.4094, *p* = 0.0097) (Fig. S5A). Significant positive correlations were also observed between urinary IL-1β levels and numbers of uPTECs (*r* = 0.4705, *p* = 0.0057), SA-β-gal^+^ uPTECs (*r* = 0.5086, *p* = 0.0025) and CD26^+^ uPTECs (*r* = 0.4246, *p* = 0.0138) (Fig. S5B). No significant correlations with patient kidney function (eGFR) at time of biopsy were identified (Fig. S5C). Collectively, these data identify a novel pathobiological mechanism in which the hypoxic-inflammatory microenvironment of the fibrotic tubulointerstitium drives senescence of PTECs that are detectable in exfoliated urinary cells.

## Discussion

Hypoxia and inflammatory IL-1β are independent mediators of tubulointerstitial fibrosis, the pathological hallmark of CKD. Here, we demonstrate how hypoxic injury and IL-1β/IL-1RI signalling act in combination to induce: (i) PTEC senescence via cell cycle arrest in G2/M phase; and (ii) a potential pro-fibrogenic (TGF-β1/TGFβR1) signalling cascade with capacity to mediate tubulointerstitial fibrosis in human CKD.

In response to hypoxic kidney injury, PTEC-derived danger signals selectively trigger the release of IL-1β by activated myeloid cells (e.g., macrophages, DCs). We have previously identified CD1c^+^ DC as a key myeloid cell source of IL-1β in human CKD, mediated via complex NLRP3 inflammasome-dependent interactions with damaged PTEC within the hypoxic tubulointerstitium [12]. Furthermore, we have detected increased levels of IL-1β in the supernatant of dissociated human fibrotic kidney biopsies compared with control kidney tissue [5]. Subsequent downstream IL-1β signalling of kidney parenchymal and immune-infiltrating cells occurs via the primary cell surface receptor, IL-1RI, or inhibitory/decoy receptor, IL-1RII [29]. IL-1β/IL-1R signalling in the diseased kidney is complex, with both protective and detrimental roles observed in discrete kidney cell lineages [30]. In a unilateral ureteral obstruction (UUO) mouse model of hypoxic CKD, IL-1RI expression is induced in tubular and interstitial cells [31], with IL-1β/IL-1RI pathway activation in tubular epithelial cells mediating progressive tubulointerstitial fibrosis [32]. Our study extends these findings to the hypoxic microenvironment of human CKD. We demonstrate the selective induction of IL-1RI, but not decoy receptor IL-1RII, in our in vitro hypoxic PTECs cultured with IL-1β and also in situ on AQP-1^+^ PTECs within human fibrotic kidneys. Previous RNA-seq analysis of human fibrotic kidney tissue assigned a prominent role for IL-1β/IL-1R signalling in CKD progression [13]. Here, we map the pathological processes driven by IL-1β/IL-1R signalling to PTECs within the hypoxic CKD microenvironment.

Experimental mouse models of kidney damage and fibrosis link IL-1β/IL-1R signalling with accelerated tubular cell senescence/arrest, with proximal tubular cells being the predominant senescent cell type [33, 34]. In particular, the accumulation of senescent PTECs arrested in the G2/M phase of the cell cycle promotes a pro-fibrogenic (e.g., TGF-β1-producing) secretory phenotype and fibrotic maladaptive repair [10, 35, 36]. We extend these studies to show that IL-1β/IL-1RI signalling in hypoxic human primary PTECs triggers hallmarks of G2/M checkpoint exit and senescence, including: (i) increased expression of p21 (cyclin-dependent kinase inhibitor 1 A; CDKN1A); (ii) G2/M cell cycle arrest (reduced expression of cyclin-dependent kinase 1 (CDK1)-associated A- and B-type cyclins, increased percentage of G2/M phase cells and decreased proliferation) [37]; (iii) lysosomal dysfunction (up-regulation of lysosomal SA-β-gal activity) [38]; (iv) destabilization of the nuclear envelope (loss of lamin B1) [24]; and (v) secretion of pro-inflammatory and pro-fibrotic SASP components (elevated IL-6 production) [11, 39]. Of note, IL-1β treatment induced similar levels of TGF-β1 in both our normoxic and hypoxic PTECs. However, TGFβR1 expression was up-regulated in only Hypoxia+IL-1β-treated PTECs, providing evidence for a potential autocrine TGF-β1/TGFβR1 signalling loop restricted to hypoxic-inflammatory conditions and capable of stimulating epithelial-to-mesenchymal transition (EMT) and tubulointerstitial fibrosis.

Senolytics are a class of drugs that selectively clear senescent cells [40]. The senolytic drug combination of quercetin and dasatinib (Q + D) has been shown to alleviate cellular senescence and kidney fibrosis in a mouse model of chronic ischaemic kidney injury [41] and reduce senescent cell burden and circulating SASP factors in a clinical trial of patients with diabetic CKD (NCT02848131) [25]. Our study confirms the senolytic efficacy of the Q + D drug combination on Hypoxia+IL-1β-treated PTECs (i.e., reduced SA-β-gal activity and IL-6 production) and establishes a robust model of human primary tubular cell senescence for future pre-clinical screening of novel senolytic therapeutics.

We have previously detected elevated levels of IL-1β protein in human hypoxic/fibrotic kidneys [5]. Our present in situ analysis of fibrotic kidney tissue further demonstrates an increase in SA-β-gal^+^ PTECs, supporting the concept of an IL-1β/hypoxic-mediated mechanism of tubular senescence induction in human CKD. Tubular SA-β-gal activity has been associated with accelerated loss of kidney function in CKD patients with different etiologies [42], although our study is the first to establish tubular segment specificity using the fluorescent Spider-β-gal probe and co-staining for PTEC marker AQP-1. Further in situ assessments are required to demonstrate co-localisation of IL-1β protein with SA-β-gal^+^ PTECs – however, IF microscopy detection of this proinflammatory cytokine in kidney tissue is technically challenging due to its dynamic secretion and short half-life [43, 44].

We and others [45] have proposed using exfoliated urinary PTECs as a non-invasive, ex vivo representation of the in situ CKD microenvironment. Here, we identify increased numbers of senescent PTECs (SA-β-gal^+^ or CD26^+^ cells) in the urine of patients with tubulointerstitial fibrosis. In particular, we associate numbers of senescent urinary PTECs with both levels of urinary IL-1β and the histological severity of CKD (levels of fibrosis). This flow cytometry-based assessment of urinary PTECs has the potential to be developed as a non-invasive assay of fibrotic CKD, with the possibility of grading histological severity (e.g., senescent cell burden and degree of interstitial fibrosis) and tracking response to CKD treatments (e.g., senolytic therapy) in real time.

Our collective results uncover a complex maladaptive signalling loop linking innate immunity with PTEC senescence in the hypoxic-inflammatory conditions of human CKD. We propose that IL-1β/IL-1RI signalling during hypoxic kidney injury drives: (i) the accumulation of senescent PTECs that (ii) release a repertoire of pro-inflammatory/fibrotic SASP (e.g. IL-6/TGF-β1) and, in turn, (iii) trigger highly sensitized TGF-βR1^+^ PTECs to mediate tubulointerstitial fibrosis. Our findings open up novel approaches to precision targeting in CKD management, including innovative therapeutics (e.g., combination therapy blocking both hypoxia and IL-1RI signalling) and diagnostic assays (e.g., use of senescent PTECs as urinary biomarkers).

## Supplementary information

Supplementary Material

Supplementary Data S1

Supplementary Data S2

Supplementary Data S3

## Data Availability

RNA-seq data (FASTQ files) supporting the findings of this study have been deposited at the European Genome-Phenome Archive (EGA) https://ega-archive.org under Accession Number EGAS00001007904. Processed RNA-seq data (count matrix and DEGs) are available in Supplementary Data S1 and Supplementary Data S2 (Tables S5-S10).
