# Supplementary material for: Human proximal tubular epithelial cell interleukin-1 receptor signalling triggers G2/M arrest and cellular senescence during hypoxic kidney injury
Source: Cell Death Dis. 2025 Jan 31;16(1):61. doi: 10.1038/s41419-025-07386-6 (PMC11785723; doi:10.1038/s41419-025-07386-6)
Supplement: Supplementary file 1 — Supplementary Material [file 41419_2025_7386_MOESM1_ESM.pdf]

SUPPLEMENTARY MATERIAL

Human proximal tubular epithelial cell interleukin-1 receptor signalling triggers G2/M arrest and cellular senescence during hypoxic kidney injury.

Authors: Kurt T.K. Giuliani[1,2,3,†], Purba Nag[1,2,†], Benjamin C. Adams[1,2,3,†], Xiangju Wang[1,2], Seokchan Hong[1,2,4], Anca Grivei[1,2], Rebecca L. Johnston[5], Nicola Waddell[3,5], Kenneth K.C. Ho[1,3], Yilin Tian[1,3], Muhammad Ali Khan[1,2,3,6,7], Chang Seong Kim[1,2,8,9], Monica S.Y. Ng[1,2,3], Glenda Gobe[3,6], Jacobus P.J. Ungerer[1,3], Josephine M. Forbes[3,10], Helen G. Healy[1,2,3,*], Andrew J. Kassianos[1,2,3,11,*].

Affiliations: [1]Conjoint Internal Medicine Laboratory, Chemical Pathology, Pathology Queensland, Brisbane, Queensland, Australia; [2]Kidney Health Service, Royal Brisbane and Women's Hospital, Brisbane, Queensland, Australia; [3]Faculty of Medicine, University of Queensland, Brisbane, Queensland, Australia; [4]Division of Rheumatology, Department of Internal Medicine, Asan Medical Centre, Seoul, Republic of Korea; [5]QIMR Berghofer Medical Research Institute, Brisbane, Queensland, Australia; [6]Kidney Disease Research Collaborative, Princess Alexandra Hospital and University of Queensland, Translational Research Institute, Brisbane, Queensland, Australia; [7]Department of Pharmacy, Bangabandhu Sheikh Mujibur Rahman Science and Technology University, Bangladesh; [8]Department of Internal Medicine, Chonnam National University Hospital, Gwangju, Republic of Korea; [9]Department of Internal Medicine, Chonnam National University Medical School, Gwangju, Republic of Korea; [10]Mater Research Institute, University of Queensland, Brisbane, Queensland, Australia; [11]Institute of Health and Biomedical Innovation/School of Biomedical Sciences, Queensland University of Technology, Brisbane, Queensland, Australia.

SUPPLEMENTARY METHODS

Bulk RNA-seq, data processing and analysis

Total RNA was isolated using the RNeasy Mini kit (QIAGEN, Hilden, Germany) according to the manufacturer's instructions. RNA was isolated from four treatment groups (Normoxia Vehicle, Normoxia IL-1β, Hypoxia Vehicle, Hypoxia IL-1β) with four biological replicates (Patients 1, 2, 3, 4 in Table S1) per group (16 samples total). Total RNA concentration and integrity were determined by RNA ScreenTape Analysis (Agilent, Santa Clara, CA, USA), with RNA integrity scores of ≥8 for all samples. Messenger RNA (mRNA) was isolated using a NEBNext® Poly(A) mRNA Magnetic Isolation kit (New England Biolabs, Ipswich, MA, USA), with directional RNA library preparation (fragmentation, cDNA synthesis, end repair/dA-tailing, adaptor ligation, PCR enrichment steps) performed using the NEBNext® Ultra™ II Directional RNA Library Prep Kit with Sample Purification Beads (New England Biolabs), along with NEBNext® multiplex oligos (Index Primers Set 1 + 2; New England Biolabs). Samples were sequenced using the NextSeq 550 (Illumina, San Diego, CA, USA) with the NextSeq 500/550 High Output v2.5 75 cycle kit (Illumina, 1 x 75 bp), with an average of 34.36 million reads (range 31.43 to 37.53 million reads) obtained per sample.

Sequence reads were trimmed for adaptor sequences using Cutadapt (version 1.9)[S1] and aligned using STAR (version 2.5.2a)[S2] to the GRCh37 assembly with the gene, transcript and exon features of the Ensembl (release 70) gene model. Quality control was performed using RNA-SeQC (version 1.1.8).[S3] Expression was estimated using RSEM (version 1.2.30).[S4] Downstream analysis was performed in R (version 4.2.0)[S5] using the expected gene counts file output by RSEM. Differential expression analysis was performed using edgeR's quasi-likelihood pipeline (version 3.40.2).[S6-8] First, protein-coding genes were selected based on Ensembl gene biotype, then a `DGEList` object was created with the `group` argument

specified as a factor (one of four treatment groups for each sample). Subsequently, the `filterByExpr` function was run using defaults to only analyse genes with sufficiently large counts, before calculating normalization factors using the `calcNormFactors` function. Log_2 counts per million (CPM) were obtained for visualization purposes using the `cpm` function with `log = TRUE`. Principal component analysis (PCA) was performed using the `prcomp` function, with transposed log_2 CPM values as input, and both `center = TRUE` and `scale. = TRUE`. A means model was fit to the data using a design matrix that incorporated both treatment group and donor without an intercept term. Namely, the design matrix was coded as `model.matrix(0 + Group + Donor)`, where Group represented the four conditions (Normoxia Vehicle, Normoxia IL-1β, Hypoxia Vehicle, Hypoxia IL-1β), and donor represented the four human primary PTEC donors. Dispersion estimates for each gene were obtained using `estimateDisp`, then quasi-likelihood dispersions were estimated using `glmQLFit`. Pairwise comparisons were created using the `makeContrasts` function from limma (version 3.54.2).[S9] To test for differential expression relative to a log-fold-change threshold, we used the `glmTreat` function with `lfc = log2(2)`. Differentially expressed genes (DEGs) between treatment groups were determined based on false discovery rate (FDR) <0.05 (`p.adjust` function using `method = "fdr"`). Mean difference plots and scatterplots were created using ggplot2 (version 3.4.2). To identify significant DEGs 'unique' to the Hypoxia IL-1β vs Normoxia Vehicle contrast, the genes with FDR <0.05 only in the Hypoxia IL-1β vs Normoxia Vehicle contrast were determined using `dplyr` (version 1.1.2); i.e. the same genes obtain FDR >0.05 in all other contrasts. These 'unique' DEGs were split into up-regulated and down-regulated DEGs (based on logFC > 0 or < 0, respectively), before performing overrepresentation analysis of Kyoto Encyclopaedia of Genes and Genomes (KEGG, https://www.genome.jp/kegg/) pathways using clusterProfiler (version 4.6.2).[S10] First, Ensembl IDs were converted to

Entrez IDs using `bitr` with `OrgDb = "org.Hs.eg.db"`, then KEGG enrichment analysis was performed using `enrichKEGG`, and dotplots were created using the `dotplot` function from enrichplot (version 1.18.4). Heatmaps were created using ComplexHeatmap (version 2.14.0), where z-scores for genes of interest were input using the `scale` function. UpSet plot was created using the UpSetR package (version 1.4.0).

Gene set enrichment analysis was performed using GSEA desktop version 4.3.3. The expression (Gene Cluster Text, gct) and phenotype (Categorical Class, cls) data files were created in R as per the GSEA User Manual. The normalized expression values output from edgeR's cpm function with `log = FALSE` were used to create the gct file. The Reactome gene sets "REACTOME_CELLULAR_SENESCENCE" (197 genes) and "REACTOME_SENESCENCE_ASSOCIATED_SECRETORY_PHENOTYPE_SASP" (111 genes) were downloaded from the human Molecular Signatures Database C2 collection (MSigDB, https://www.gsea-msigdb.org/gsea/msigdb) in Gene Matrix Transposed (gmt) format. After loading the gct, cls and gmt files into GSEA, a standard GSEA analysis was performed using the default parameters, except for `Permutation type = gene_set` and `Seed for permutation = 42`. Enrichment plots were created using the replotGSEA function from Rtoolbox version 1.4 (https://github.com/PeeperLab/Rtoolbox/).

Protein expression by Western blotting

Primary PTECs were lysed with urea lysis buffer and protein concentration determined using the Pierce™ bicinchoninic acid (BCA) protein assay kit (Thermo Fisher Scientific, Waltham, MA, USA). Polyacrylamide gel electrophoresis (PAGE) was undertaken using standard reagents from Thermo Fisher Scientific. Samples were denatured for 5 min at 95°C, loaded onto Bolt™ 4-12% Bis-Tris Plus Gels, run at 200V for 22 min and transferred to a

nitrocellulose membrane at 10V for 60 min. Membranes were blocked for 1 hour at room temperature with Intercept (TBS) blocking buffer (LI-COR, Lincoln, NE, USA) and subsequently probed with primary antibodies overnight at 4°C, including transforming growth factor β receptor 1 (TGFβR1) (1:500; Rabbit monoclonal IgG; Cat. No. ab235578; Abcam, Cambridge, MA, USA), Cyclin A2 (1:1000; Rabbit monoclonal IgG; Cat. No. 67955; Cell Signaling Technology, Danvers, MA, USA), p21 (1:500; Mouse monoclonal IgG; Cat. No. 556431; BD Biosciences, San Jose, CA, USA) and β-actin (1:1000; Mouse monoclonal IgG; Cat. No. ab8224; Abcam). Proteins were visualized with IRDye 800CW goat anti-mouse (1:15,000; Millennium Science, Mulgrave, VIC, Australia) or IRDye 680LT goat anti-rabbit (1:20,000; Millennium Science) using the Odyssey CLX (LI-COR). Quantitative analysis of protein intensities relative to β-actin loading control was performed using Image Studio 5.2 software (LI-COR).

Protein expression by flow cytometry

Harvested primary PTECs were stained with LIVE/DEAD® Fixable Near-IR Dead Cell Stain Kit (Life Technologies, Grand Island, NY, USA) as per manufacturer's recommendations to assess cell viability. Cells were then incubated with Human TruStain FcX™ Blocking Solution (Biolegend, San Diego, CA, USA) at room temperature for 5-10 min and then stained on ice for 20-30 min with a combination of test (IL-1RI-APC (Cat. No. FAB269A100) and IL1RII-Alexa Fluor™ 700 (Cat. No. FAB663N); both from R&D Systems, Minneapolis, MN, USA) or isotype-matched control antibodies (R&D Systems) in cold FACS buffer (0.5% bovine serum albumin (BSA; Sigma-Aldrich, St Louis, MO, USA) and 0.02% sodium azide (Sigma-Aldrich) in PBS). Cell acquisition was performed on an LSR Fortessa (BD Biosciences) and data analyzed with FlowJo software (TreeStar, Ashland, OR, USA).

Cell cycle analysis by flow cytometry

Cell cycle distribution was examined using the Cell Cycle Analysis kit (Abcam). Briefly, harvested PTECs were fixed in 70% ethanol at 4°C overnight, followed by staining at room temperature for 30 min in the dark with propidium iodide (to measure DNA content that distinguishes phases of the cell cycle) and RNase A (to remove contaminating RNA signal). Cell acquisition was performed on an LSR Fortessa (BD Biosciences), with data analyzed using FlowJo software (TreeStar) and validated with ModFit LT software (Verity Software House, Topsham, ME, USA).

Cell proliferation measurements of human primary PTECs

Cell proliferation was investigated using the colorimetric MTT (3-(4,5-dimethylthiazol-2-yl)-2,5-diphenyltetrazolium bromide) Cell Proliferation Assay kit (Molecular Probes, Eugene, OR, USA). PTECs were seeded (20,000 cells/well in DM) in triplicate in 96-well flat-bottom plates to allow overnight adherence and then cultured for a further 72 hours in fresh, conditioned DM under normoxic or hypoxic conditions in the absence or presence of 1ng/ml recombinant human IL-1β. Culture supernatants were harvested following the 72 hour treatment period and levels of soluble proteins were determined using the LEGENDplex™ Human Essential Immune Response Panel multiplex bead array (BioLegend) following the manufacturer's recommendations. MTT solution (10µl of 12mM stock) was administered to PTECs, followed by a 2.5 hour incubation at 37°C. The MTT-containing medium was subsequently removed and dimethyl sulfoxide (DMSO; Sigma-Aldrich) applied to the cells, followed by a 10 min incubation at 37°C. Absorbance values at 540nm were determined using a Powerwave X52 microplate reader (BioTek Instruments, Winooski, VT, USA).

Senescence associated β-galactosidase (SA-β-gal) staining of human primary PTECs

SA-β-gal activity was determined using the Senescence β-Galactosidase Staining Kit (Cell Signaling Technology) following the manufacturer's instructions. Briefly, cells were washed with PBS, fixed with 1x Fixative Solution for 15 min at room temperature, washed 3x with PBS and incubated overnight at 37°C with the 1mg/ml X-gal staining solution (pH 6.0). The cells were washed once with PBS before coverslips were mounted on slides in fluorescence mounting medium (Dako Omnis; Agilent Technologies) and imaged using an EVOS® FL Auto Imaging System (Thermo Fisher Scientific) at 10x magnification. Image processing and analysis was performed using FIJI software (version 2.35),[S11] with mean percentage of positive cells calculated from five random fields of view for each sample.

SUPPLEMENTARY FIGURES

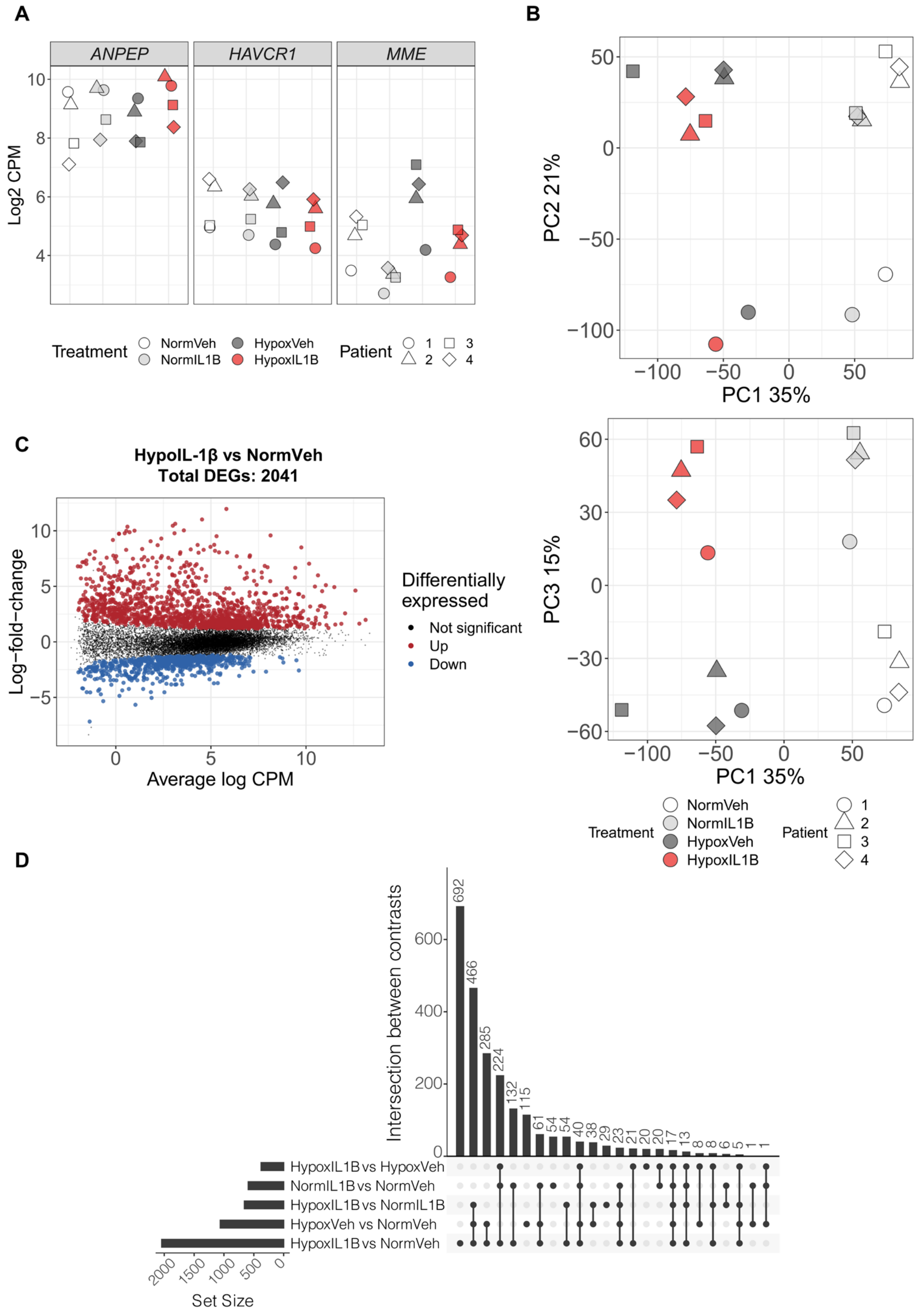

Supplementary Figure S1. RNA-seq reveals 'uniquely' differentially expressed genes (DEGs) in Hypoxia+IL-1β-treated versus Normoxia+Vehicle PTECs. (A) Scatterplot of normalized expression values ($\log_2$ CPM) for established PTEC markers *ANPEP* (CD13), *HAVCR1* (KIM-1) and *MME* (CD10) across all PTEC samples. **(B)** Principal Component Analysis (PCA) of all protein-coding genes that passed minimum expression filter.

Normalized expression values (scaled and centred) were used as input to PCA. Top panel: PC1 vs PC2; lower panel: PC1 vs PC3. **(C)** Mean difference (MD) plot of log_2 fold change vs log_2 CPM for the Hypoxia+IL-1β vs Normoxia+Vehicle contrast, with significantly (FDR <0.05) up-regulated (logFC >0) genes shaded red and significantly down-regulated (logFC <0) genes shaded blue. **(D)** UpSet plot displaying the intersection of DEGs between all five contrasts. Dots with connecting lines indicate contrasts with common DEGs, and dots with no connecting lines indicate DEGs unique to the contrast. Comparison identified 692 DEGs unique to the Hypoxia+IL-1β vs Normoxia+Vehicle contrast.

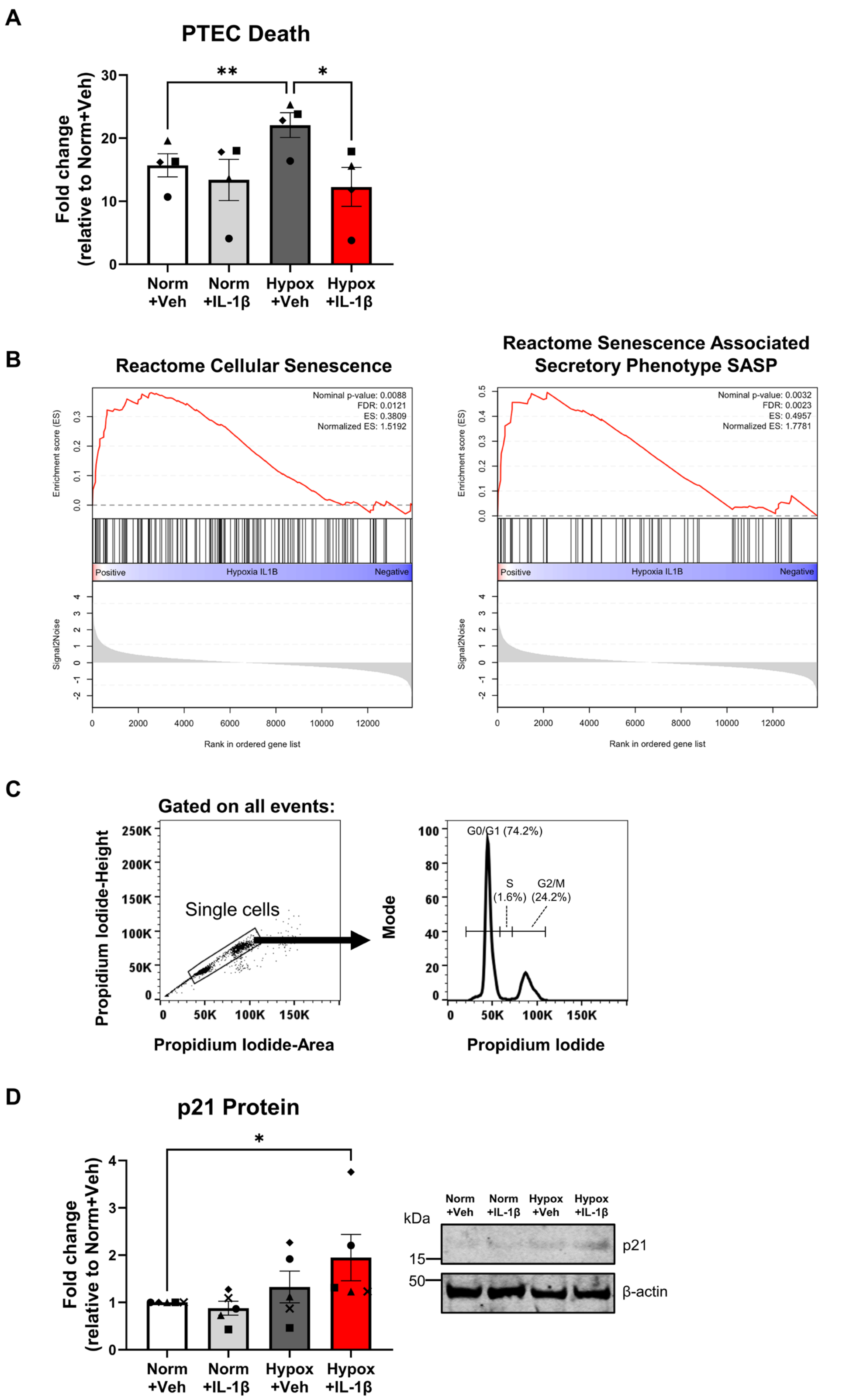

Supplementary Figure S2. Molecular profiling reveals cellular senescence gene sets and protein biomarkers are enriched in Hypoxia+IL-1β-treated PTECs. (A) Fold changes (relative to Normoxia+Vehicle) in cell death (% Near-IR viability dye^{+} cells, gated on all

single events) for PTECs cultured for 48 hours under normoxic (norm) or hypoxic (hypox) conditions in the absence (vehicle; veh) or presence of IL-1β. Bar graphs represent mean ± SEM. Symbols represent individual donor PTECs; n=4. *P<0.05, **P<0.01, one-way ANOVA with Tukey's multiple-comparison test. **(B)** Gene set enrichment analysis (GSEA) results for gene sets Reactome Cellular Senescence (left panel) and Reactome senescence associated secretory phenotype SASP (right panel) in Hypoxia+IL-1β versus Hypoxia+Vehicle PTECs. **(C)** Gating strategy for cell cycle analysis as per manufacturer's recommendations: Initial gating of single cells in the PI-Area vs PI-Height plot was applied to exclude debris/dead cells and aggregates/doublets (left panel), followed by cell cycle analysis to calculate percentages of G0/G1, S and G2/M phase cells (right panel). **(D)** Left panel: Fold changes (relative to Normoxia+Vehicle) in p21 protein levels (as a ratio of loading control β-actin) for PTECs cultured for 48 hours under normoxic (norm) or hypoxic (hypox) conditions in the absence (vehicle; veh) or presence of IL-1β. Bar graphs represent mean ± SEM. Symbols represent individual donor PTECs; n=5. *P<0.05, one-way ANOVA with Tukey's multiple-comparison test. Right panel: p21 Western blot for PTECs cultured under normoxic (norm) or hypoxic (hypox) conditions in the absence (vehicle; veh) or presence of IL-1β (20μg total protein per lane). Representative images from one of five donor PTECs are presented. Full and uncropped Western blot available as Supplementary Material.

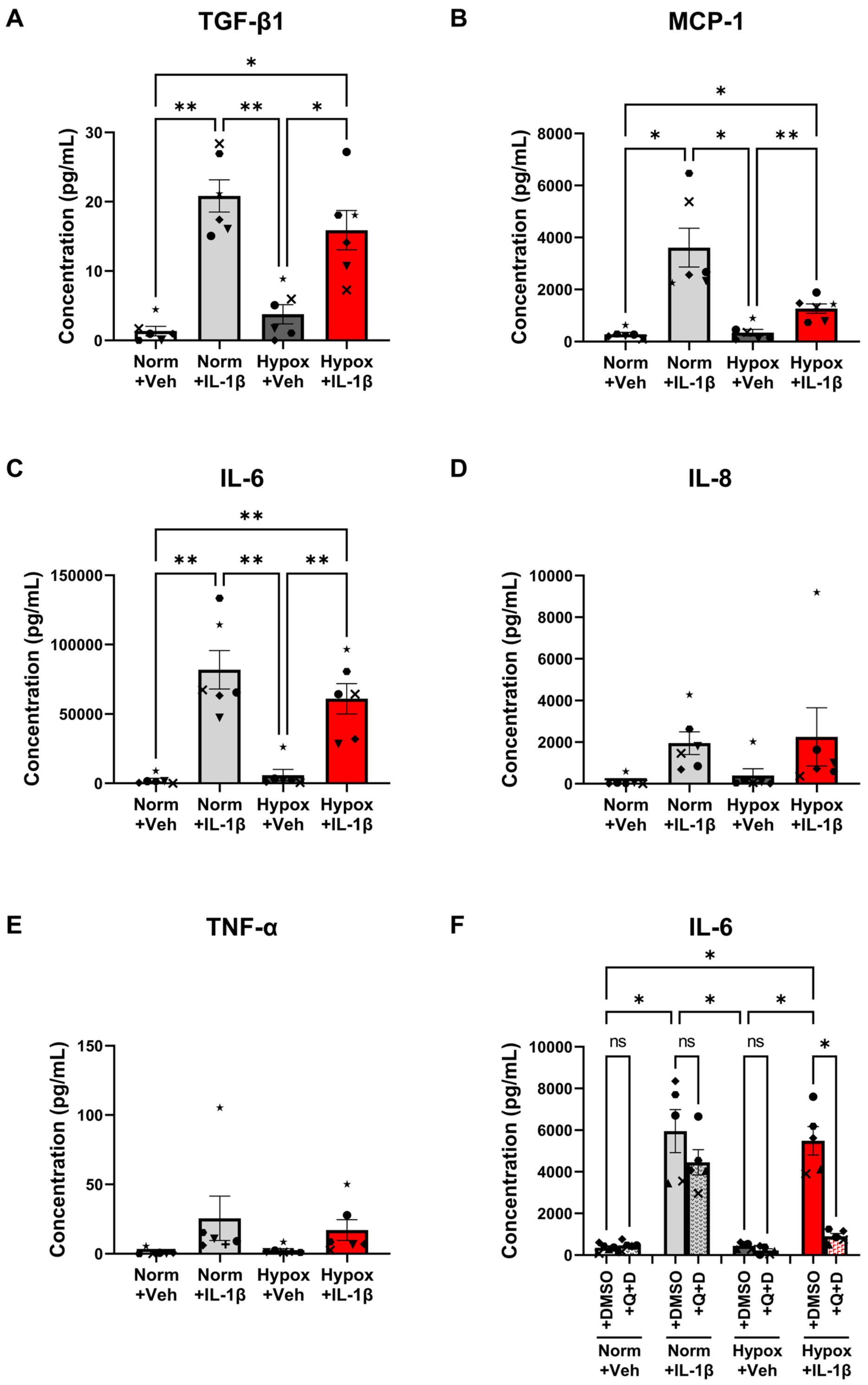

Supplementary Figure S3. Screening of senescence-associated secretory phenotype (SASP) factors in PTEC culture supernatants. (A-E) Transforming growth factor (TGF)-β1 **(A)**, monocyte chemoattractant protein (MCP)-1 **(B)**, interleukin (IL)-6 **(C)**, IL-8 **(D)** and

tumour necrosis factor (TNF)-α **(E)** protein levels (measured by LEGENDplex™ assay; pg/ml) following 72 hour culture of human primary PTECs under normoxic (norm) or hypoxic (hypox) conditions in the absence (vehicle; veh) or presence of IL-1β. Bar graphs represent mean ± SEM. Symbols represent individual donor PTECs; n=6. *$P<0.05$, **$P<0.01$, one-way ANOVA with Tukey's multiple-comparison test. IL-2, IL-4, IL-10, IL-12p70, IL-17A, interferon (IFN)-γ and IFN-γ-induced protein 10 (IP-10) were not detectable in any culture conditions (data not shown). **(F)** IL-6 protein (measured by LEGENDplex™ assay; pg/ml) produced by human primary PTECs initially cultured under normoxic (norm) or hypoxic (hypox) conditions in the absence (vehicle; veh) or presence of IL-1β, followed by additional 48-hour treatment in fresh DM without (DMSO vehicle control) or with quercetin+dasatinib (Q+D) for cytokine screening. Bar graphs represent mean ± SEM. Symbols represent individual donor PTECs; n=5. *$P<0.05$, one-way ANOVA with Tukey's multiple-comparison test; ns, not significant.

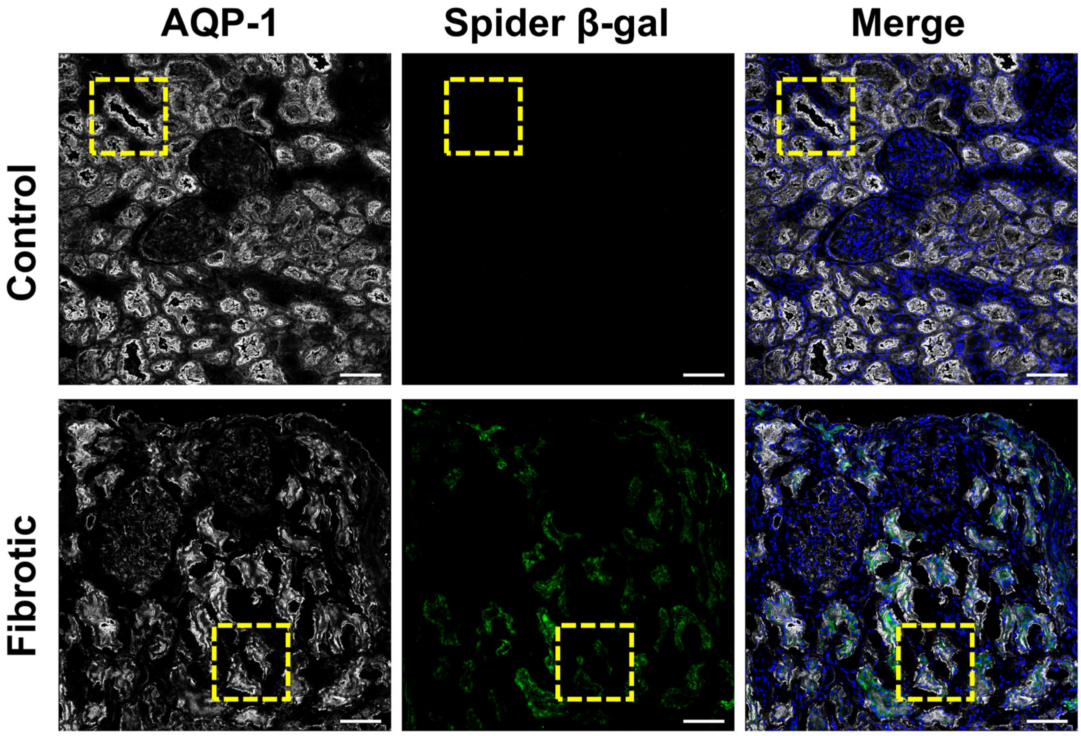

Supplementary Figure S4. PTEC senescence in fibrotic kidneys. Representative immunofluorescent images of control (non-fibrotic) and fibrotic kidney tissue labelled with PTEC marker AQP-1 (white), Spider-β-gal (green) and DAPI (blue). Scale bars represent 100μm. Higher magnification regions presented in Figure 4 are highlighted with yellow (dashed) boxes.

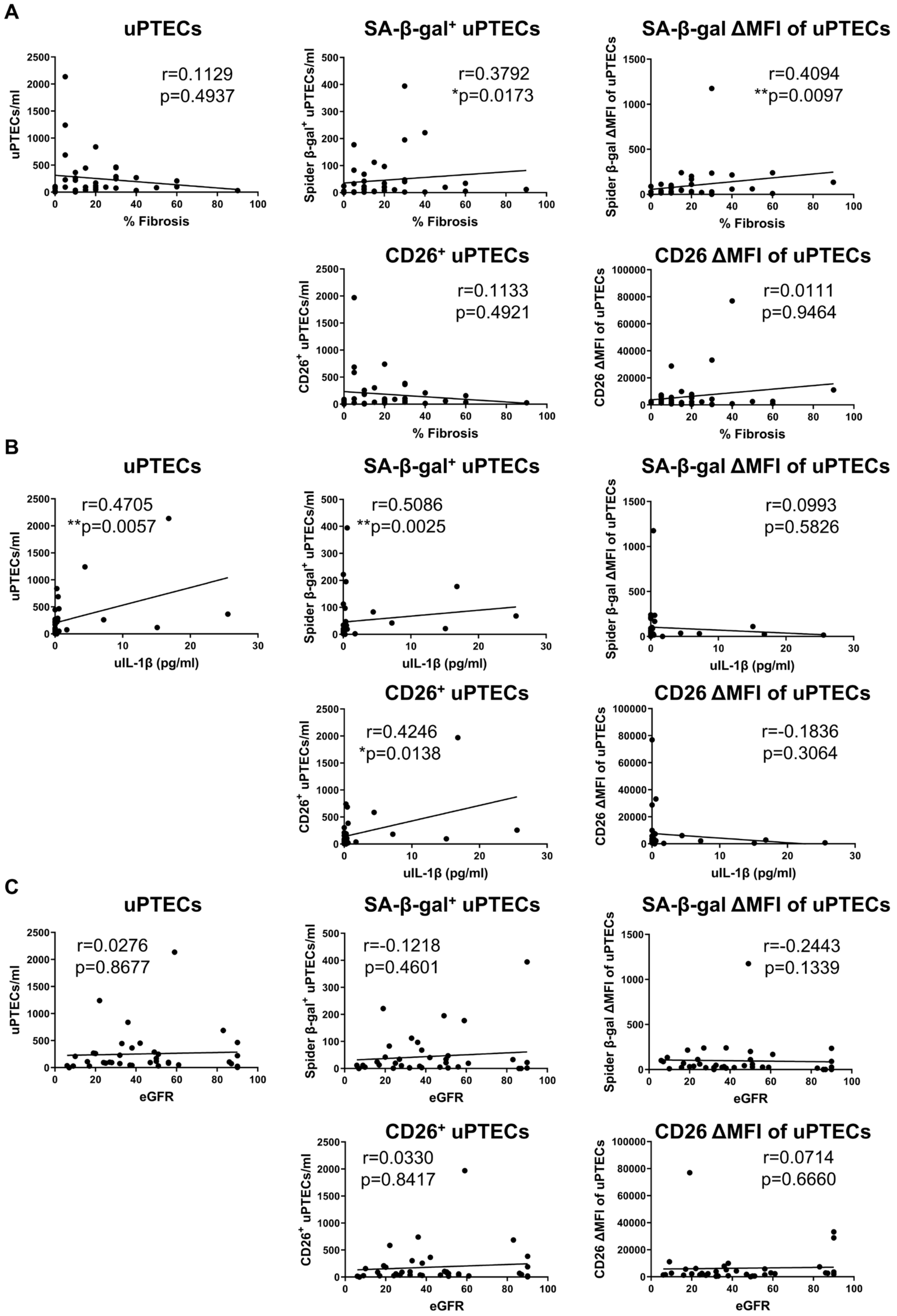

Supplementary Figure S5. Senescent urinary PTEC numbers correlate significantly with the degree of interstitial fibrosis and urinary IL-1β levels. (A-C) Spearman correlation analysis of numbers of urinary PTECs (uPTECs/ml urine), numbers of SA-β-gal^{+} uPTECs (SA-β-gal^{+} uPTECs/ml urine), Spider-β-gal fluorescence intensity of uPTECs

(ΔMFI), numbers of $CD26^+$ uPTECs ($CD26^+$ uPTECs/ml urine) and CD26 fluorescence intensity of uPTECs (ΔMFI) versus percentages of interstitial fibrosis in contemporaneous kidney biopsies (% fibrosis) (n=39) **(A)**, levels of urinary IL-1β (uIL-1β; pg/ml urine) (n=33) **(B)** and patient kidney function (estimated glomerular filtration rate; eGFR) (n=39) **(C)**.

SUPPLEMENTARY TABLES

Supplementary Table S1. Clinical and histological features of PTEC donors at the time of nephrectomy. [a]RCC – Renal cell carcinoma; [b]Kidney function (estimated glomerular filtration rate; eGFR) calculated using the CKD-EPI equation; *Selected for bulk RNA-seq analysis.

Patient	Age (Years)/ Sex (M/F)	Symbol	Primary Disease	eGFR (ml/min/1.73m^2)[b]
1*	45/M	●	Clear cell RCC[a] (Grade 2)	>90
2*	65/M	▲	Clear cell RCC (Grade 3)	>90
3*	52/M	■	Clear cell RCC (Grade 2)	>90
4*	63/M	◆	Clear cell RCC (Grade 2)	77
5	60/M	✚	Clear cell RCC (Grade 3)	84
6	55/M	▼	Clear cell RCC (Grade 2)	88
7	59/M	⬣	RCC (Epithelioid tumour)	>90
8	58/M	x	Clear cell RCC (Grade 2)	81
9	61/M	★	Clear cell RCC (Grade 2)	88

Supplementary Table S2. Clinical and histological features of patients at the time of urine specimen collection/kidney biopsy. [a]Kidney function (estimated glomerular filtration rate; eGFR) calculated using the CKD-EPI equation; *No urinary supernatant available for matching urinary IL-1β detection.

Patient	Age (Years)/ Sex (M/F)	Primary Disease	eGFR (ml/min/1.73m²)[a]	Tubulointerstitial Fibrosis (%)
Control/Non-fibrotic (n=7)				
1	68/F	Cresentic glomerulonephritis	56	0
2	28/M	Membranous nephropathy	90	0
3	37/M	IgA nephropathy	90	0
4	72/F	Membranous nephropathy	87	0
5	28/F	Lupus nephritis	86	0
6	44/F	Interstitial nephritis	7	0
7*	34/M	Interstitial nephritis	16	0
Fibrotic (n=32)				
1	60/F	Diabetic nephropathy	51	30
2	51/M	Focal segmental glomerulosclerosis	61	20
3*	70/M	Diabetic nephropathy	24	5
4	64/F	Focal segmental glomerulosclerosis	90	10
5	68/M	Cresentic glomerulonephritis	6	10
6	62/M	IgA nephropathy	47	15
7*	35/M	Diabetic nephropathy	9	90
8*	27/F	Diabetic nephropathy	51	40
9	54/F	IgA nephropathy	50	20
10	47/M	Thrombotic microangiopathy	10	60
11	28/F	Diabetic nephropathy	56	20
12	68/M	Interstitial nephritis	25	50
13	51/F	Diabetic nephropathy	17	10
14	67/F	Membranous nephropathy	83	5
15	69/M	Interstitial nephritis	28	25
16	82/F	Diabetic nephropathy	37	20
17	44/F	Arterionephrosclerosis	50	20
18*	30/F	Lupus nephritis	42	30
19	61/F	Diabetic nephropathy	32	30
20	30/M	Diabetic nephropathy	38	15
21	45/M	IgA nephropathy	90	30
22	71/F	Focal segmental glomerulosclerosis	49	30
23	71/M	IgA nephropathy	22	5
24	27/F	Membranoproliferative glomerulonephritis	59	5
25	69/M	Membranoproliferative glomerulonephritis	32	5
26*	68/F	Diabetic nephropathy	27	60
27	80/F	Membranoproliferative glomerulonephritis	20	10
28	75/M	IgA nephropathy	33	15
29	71/M	Focal segmental glomerulosclerosis	38	10
30	39/M	Membranous nephropathy	90	10
31	39/M	Diabetic nephropathy	36	20
32	31/F	Lupus nephritis	19	40

Supplementary Table S3. Antibodies used for flow cytometric staining of urinary cells.

Antigen	Clone	Fluorochrome	Source
CD45	HI30	Brilliant Violet 510	Biolegend
CD13	WM15	Brilliant Violet 785	Biolegend
CD10	HI10a	PE-Dazzle 594	Biolegend
CD26	M-A261	Brilliant Violet 650	BD Biosciences

Supplementary Table S4. Numbers of differentially expressed genes (DEGs) between PTEC treatment groups.

Contrast	Total DEGs	Up-regulated	Down-regulated
Hypoxia IL-1β - Normoxia Vehicle	2041	1364	677
Hypoxia IL-1β - Normoxia IL-1β	659	540	119
Hypoxia IL-1β - Hypoxia Vehicle	377	287	90
Hypoxia Vehicle - Normoxia Vehicle	1060	798	262
Normoxia IL-1β - Normoxia Vehicle	592	442	150

SUPPLEMENTARY DATA

Supplementary Data S1 File (xlsx)

- **Supplementary Data S1.** RNA-seq count matrix (genewise expected counts) output by RSEM.

Supplementary Data S2 File (xlsx)

- **Supplementary Table S5.** edgeR glmTreat results for Hypoxia IL-1β vs Normoxia Vehicle contrast filtered by FDR <0.05.
- **Supplementary Table S6.** edgeR glmTreat results for Hypoxia IL-1β vs Normoxia IL-1β contrast filtered by FDR <0.05.
- **Supplementary Table S7.** edgeR glmTreat results for Hypoxia IL-1β vs Hypoxia Vehicle contrast filtered by FDR <0.05.
- **Supplementary Table S8.** edgeR glmTreat results for Hypoxia Vehicle vs Normoxia Vehicle contrast filtered by FDR <0.05.
- **Supplementary Table S9.** edgeR glmTreat results for Normoxia IL-1β vs Normoxia Vehicle contrast filtered by FDR <0.05.
- **Supplementary Table S10.** Intersect of DEGs from all contrasts to find common and unique DEGs.
- **Supplementary Table S11.** enrichKEGG results for up-regulated DEGs unique to Hypoxia IL-1β vs Normoxia Vehicle contrast.
- **Supplementary Table S12.** enrichKEGG results for down-regulated DEGs unique to Hypoxia IL-1β vs Normoxia Vehicle contrast.

Supplementary Data S3 File (pdf)

- Full length uncropped original western blots for Figures 1F and 2D and Supplementary Figure S2D.

SUPPLEMENTARY REFERENCES

S1. Martin M. Cutadapt removes adapter sequences from high-throughput sequencing reads. *EMBnet.journal* **17,** 10-12 (2011).

S2. Dobin A, Davis CA, Schlesinger F, Drenkow J, Zaleski C, Jha S*, et al.* STAR: ultrafast universal RNA-seq aligner. *Bioinformatics* **29,** 15-21 (2013).

S3. DeLuca DS, Levin JZ, Sivachenko A, Fennell T, Nazaire MD, Williams C*, et al.* RNA-SeQC: RNA-seq metrics for quality control and process optimization. *Bioinformatics* **28,** 1530-1532 (2012).

S4. Li B, Dewey CN. RSEM: accurate transcript quantification from RNA-Seq data with or without a reference genome. *BMC Bioinformatics* **12,** 323 (2011).

S5. Team RC. R: A language and environment for statistical computing. 2022; Available from: https://www.R-project.org/.

S6. Robinson MD, McCarthy DJ, Smyth GK. edgeR: a Bioconductor package for differential expression analysis of digital gene expression data. *Bioinformatics* **26,** 139-140 (2010).

S7. McCarthy DJ, Chen Y, Smyth GK. Differential expression analysis of multifactor RNA-Seq experiments with respect to biological variation. *Nucleic Acids Res* **40,** 4288-4297 (2012).

S8. Chen Y, Lun AT, Smyth GK. From reads to genes to pathways: differential expression analysis of RNA-Seq experiments using Rsubread and the edgeR quasi-likelihood pipeline. *F1000Res* **5,** 1438 (2016).

S9. Ritchie ME, Phipson B, Wu D, Hu Y, Law CW, Shi W*, et al.* limma powers differential expression analyses for RNA-sequencing and microarray studies. *Nucleic Acids Res* **43,** e47 (2015).

S10. Wu T, Hu E, Xu S, Chen M, Guo P, Dai Z*, et al.* clusterProfiler 4.0: A universal enrichment tool for interpreting omics data. *Innovation (Camb)* **2,** 100141 (2021).

S11. Schindelin J, Arganda-Carreras I, Frise E, Kaynig V, Longair M, Pietzsch T*, et al.* Fiji: an open-source platform for biological-image analysis. *Nat Methods* **9,** 676-682 (2012).
