# Supplementary figures and images for: Human proximal tubular epithelial cell interleukin-1 receptor signalling triggers G2/M arrest and cellular senescence during hypoxic kidney injury
Source: Cell Death Dis. 2025 Jan 31;16(1):61. doi: 10.1038/s41419-025-07386-6 (PMC11785723; doi:10.1038/s41419-025-07386-6)

Figure 1F

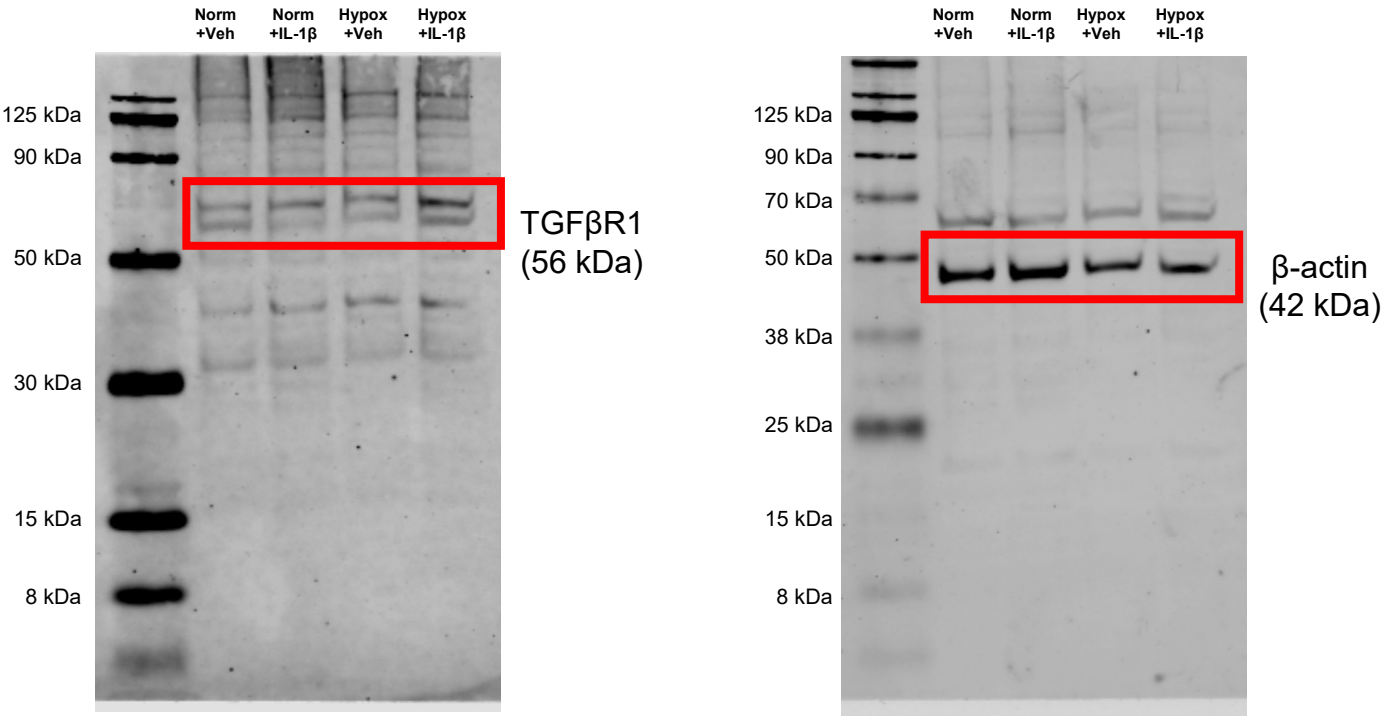

Figure 2D

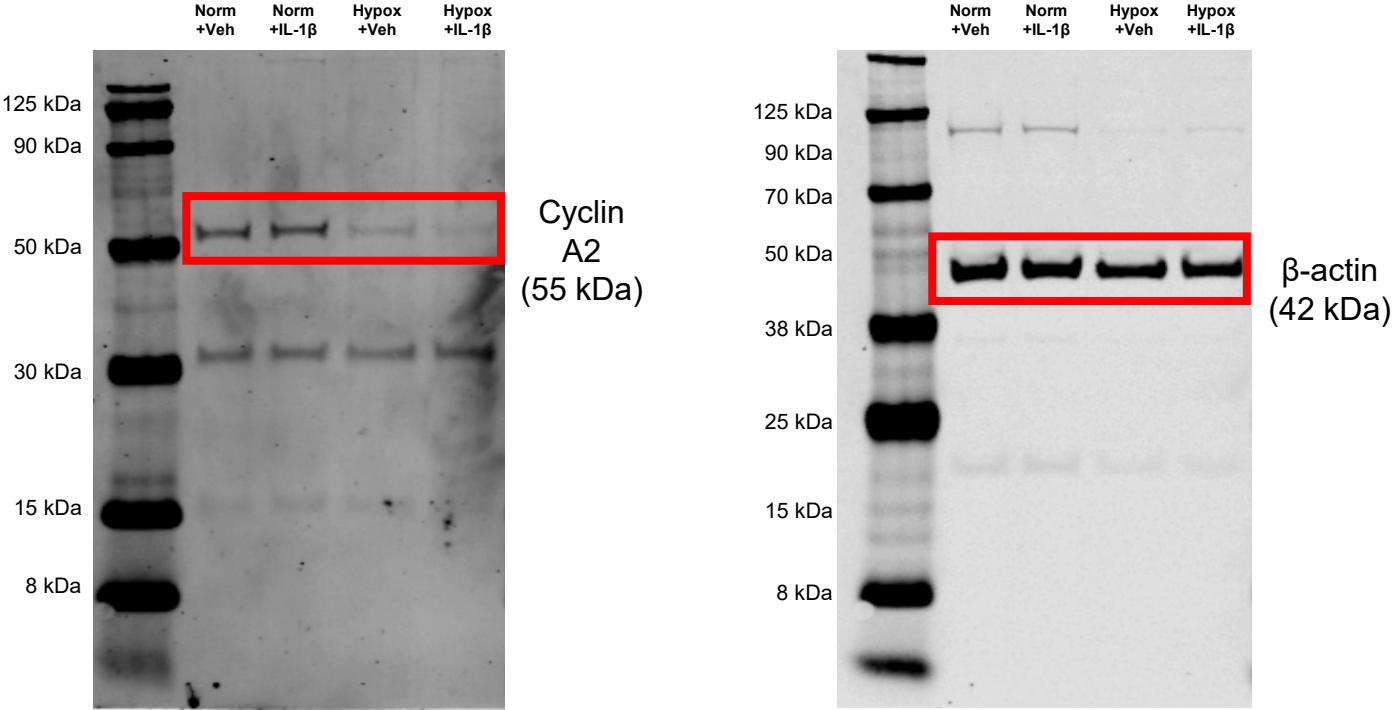

Figure S2D

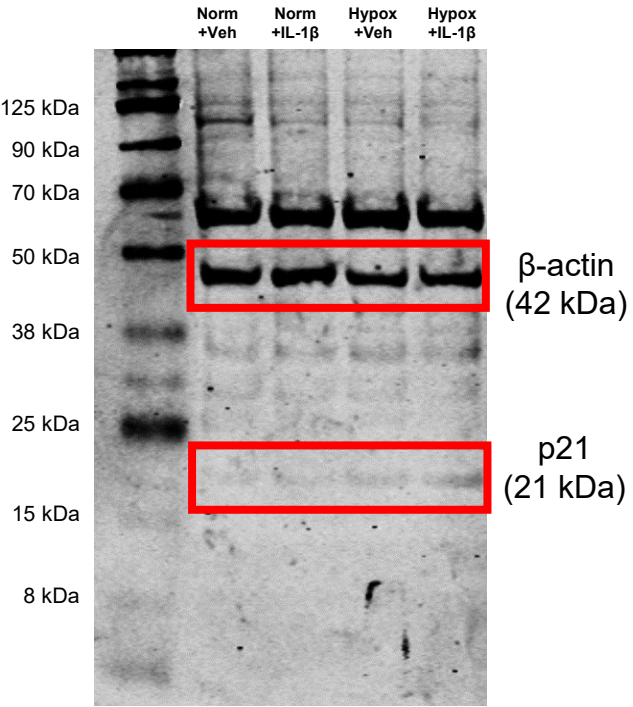

Supplement: Supplementary file 4 — Supplementary Data S3 [file 41419_2025_7386_MOESM4_ESM.pdf]
